# Prevalence, risk factors, impact and management of pneumonia among preschool children in Chinese seven cities: a cross-sectional study with interrupted time series analysis

**DOI:** 10.1186/s12916-023-02951-2

**Published:** 2023-06-26

**Authors:** Haonan Shi, Tingting Wang, Zhuohui Zhao, Dan Norback, Xiaowei Wang, Yongsheng Li, Qihong Deng, Chan Lu, Xin Zhang, Xiaohong Zheng, Hua Qian, Ling Zhang, Wei Yu, Yuqing Shi, Tianyi Chen, Huaijiang Yu, Huizhen Qi, Ye Yang, Lan Jiang, Yuting Lin, Jian Yao, Junwen Lu, Qi Yan, for the China, Children, Homes, Health (CCHH) Study Group

**Affiliations:** 1 School of Nursing & Health Management, Shanghai University of Medicine & Health Sciences, No.279, Zhouzhu Highway, Pudong New District, Shanghai, 201318 China; 2 Department of Environmental Health, School of Public Health, Fudan University, Shanghai, 200433 China; 3 Key Lab of Public Health Safety of the Ministry of Education, NHC Key Lab of Health Technology Assessment (Fudan University), Shanghai, 200433 China; 4 Department of Medical Sciences, Occupational and Environmental Medicine, Uppsala University, SE-751 Uppsala, Sweden; 5 Department of Operation and Security, Zhoupu Hospital Affiliated to Shanghai University of Medicine & Health Sciences, Shanghai, 201318 China; 6 Department of Preventive Medicine, Medical College, Shihezi University, Shihezi, 832002 China; 7 School of Public Health, Central South University, Changsha, 410083 China; 8 Research Center for Environmental Science and Engineering, Shanxi University, Taiyuan, 237016 China; 9 School of Energy and Environment, Southeast University, Nanjing, 214135 China; 10 Wuhan University of Science and Technology, Wuhan, 430081 China; 11 Joint International Research Laboratory of Green Buildings and Built Environments (Ministry of Education), Chongqing University, Chongqing, 400044 China; 12 National Centre for International Research of Low-Carbon and Green Buildings, Ministry of Science and Technology), Chongqing University, Chongqing, 400044 China; 13 People’s Hospital of Bayingguoleng Mongolian Autonomous Prefecture, Kuerle, 841099 China; 14 Department of Neurology, The Fifth Affiliated Hospital of Xinjiang Medical University, Urumqi, 830011 China; 15 Department of No.1 Cadres, The Second Affiliated Hospital of Xinjiang Medical University, Urumqi, 830063 China; 16 Department of Laboratory Medicine, Xinjiang Uyghur Autonomous Region Maternal and Child Health Hospital, Urumqi, 830001 China; 17 School of Public Health, Xinjiang Medical University, Urumqi, 830054 China; 18 Xinjiang Key Laboratory of Special Environment and Health Research, Urumqi, 830054 China

**Keywords:** Pneumonia, Preschool children, Risk factors

## Abstract

**Background:**

Pneumonia is a common disease worldwide in preschool children. Despite its large population size, China has had no comprehensive study of the national prevalence, risk factors, and management of pneumonia among preschool children. We therefore investigated the prevalence of pneumonia among preschool children in Chinese seven representative cities, and explore the possible risk factors of pneumonia on children, with a view to calling the world's attention to childhood pneumonia to reduce the prevalence of childhood pneumonia.

**Methods:**

Two group samples of 63,663 and 52,812 preschool children were recruited from 2011 and 2019 surveys, respectively. Which were derived from the cross-sectional China, Children, Homes, Health (CCHH) study using a multi-stage stratified sampling method. This survey was conducted in kindergartens in seven representative cities. Exclusion criteria were younger than 2 years old or older than 8 years old, non-permanent population, basic information such as gender, date of birth and breast feeding is incomplete. Pneumonia was determined on the basis of parents reported history of clearly diagnosed by the physician. All participants were assessed with a standard questionnaire. Risk factors for pneumonia, and association between pneumonia and other respiratory diseases were examined by multivariable-adjusted analyses done in all participants for whom data on the variables of interest were available. Disease management was evaluated by the parents’ reported history of physician diagnosis, longitudinal comparison of risk factors in 2011 and 2019.

**Results:**

In 2011 and 2019, 31,277 (16,152 boys and 15,125 girls) and 32,016 (16,621 boys and 15,395 girls) preschool children aged at 2–8 of permanent population completed the questionnaire, respectively, and were thus included in the final analysis. The findings showed that the age-adjusted prevalence of pneumonia in children was 32.7% in 2011 and 26.4% in 2019. In 2011, girls (odds ratio [OR] 0.91, 95%CI [confidence interval]0.87–0.96; *p* = 0.0002), rural (0.85, 0.73–0.99; *p *= 0.0387), duration of breastfeeding ≥ 6 months(0.83, 0.79–0.88; *p *< 0.0001), birth weight (g) ≥ 4000 (0.88, 0.80–0.97; *p* = 0.0125), frequency of putting bedding to sunshine (Often) (0.82, 0.71–0.94; *p* = 0.0049), cooking fuel type (electricity) (0.87, 0.80–0.94; *p* = 0.0005), indoor use air-conditioning (0.85, 0.80–0.90; *p* < 0.0001) were associated with a reduced risk of childhood pneumonia. Age (4–6) (1.11, 1.03–1.20; *p* = 0.0052), parental smoking (one) (1.12, 1.07–1.18; *p* < 0.0001), used antibiotics (2.71, 2.52–2.90; *p* < 0.0001), history of parental allergy (one and two) (1.21, 1.12–1.32; *p* < 0.0001 and 1.33, 1.04–1.69; *p* = 0.0203), indoor dampness (1.24, 1.15–1.33; *p* < 0.0001), home interior decoration (1.11, 1.04–1.19; *p* = 0.0013), Wall painting materials (Paint) (1.16, 1.04–1.29; *p* = 0.0084), flooring materials (Laminate / Composite wood) (1.08, 1.02–1.16; *p* = 0.0126), indoor heating mode(Central heating)(1.18, 1.07–1.30, *p* = 0.0090), asthma (2.38, 2.17–2.61; *p* < 0.0001), allergic rhinitis (1.36, 1.25–1.47; *p* < 0.0001), wheezing (1.64, 1.55–1.74; *p* < 0.0001) were associated with an elevated risk of childhood pneumonia; pneumonia was associated with an elevated risk of childhood asthma (2.53, 2.31–2.78; *p* < 0.0001), allergic rhinitis (1.41, 1.29–1.53; *p* < 0.0001) and wheezing (1.64, 1.55–1.74; *p* < 0.0001). In 2019, girls (0.92, 0.87–0.97; *p* = 0.0019), duration of breastfeeding ≥ 6 months (0.92, 0.87–0.97; *p* = 0.0031), used antibiotics (0.22, 0.21–0.24; *p* < 0.0001), cooking fuel type (Other) (0.40, 0.23–0.63; p = 0.0003), indoor use air-conditioning (0.89, 0.83–0.95; *p* = 0.0009) were associated with a reduced risk of childhood pneumonia. Urbanisation (Suburb) (1.10, 1.02–1.18; *p* = 0.0093), premature birth (1.29, 1.08–1.55; *p* = 0.0051), birth weight (g) < 2500 (1.17, 1.02–1.35; *p* = 0.0284), parental smoking (1.30, 1.23–1.38; *p* < 0.0001), history of parental asthma (One) (1.23, 1.03–1.46; *p* = 0.0202), history of parental allergy (one and two) (1.20, 1.13–1.27; *p* < 0.0001 and 1.22, 1.08–1.37; *p* = 0.0014), cooking fuel type (Coal) (1.58, 1.02–2.52; *p* = 0.0356), indoor dampness (1.16, 1.08–1.24; *p* < 0.0001), asthma (1.88, 1.64–2.15; *p* < 0.0001), allergic rhinitis (1.57, 1.45–1.69; *p* < 0.0001), wheezing (2.43, 2.20–2.68; *p* < 0.0001) were associated with an elevated risk of childhood pneumonia; pneumonia was associated with an elevated risk of childhood asthma (1.96, 1.72–2.25; *p* < 0.0001), allergic rhinitis (1.60, 1.48–1.73; *p* < 0.0001) and wheezing (2.49, 2.25–2.75; *p* < 0.0001).

**Conclusions:**

Pneumonia is prevalent among preschool children in China, and it affects other childhood respiratory diseases. Although the prevalence of pneumonia in Chinese children shows a decreasing trend in 2019 compared to 2011, a well-established management system is still needed to further reduce the prevalence of pneumonia and reduce the burden of disease in children.

**Supplementary Information:**

The online version contains supplementary material available at 10.1186/s12916-023-02951-2.

## Background

Pneumonia is a common respiratory disease worldwideamong preschool children and is the leading cause of death in children. Before 2006, more than 2 million children younger than 5 years were reported to die of pneumonia each year, accounting for one-fifth of all deaths in children younger than 5 years worldwide [1]. Among them, developing Asia–Pacific countries have a higher prevalence of pneumonia in children [2]. To illustrate, in 2008, there were approximately 43 million and 10 million pediatric patients with pneumonia under 5 years of age in India and Pakistan, respectively [3]. There has been no comprehensive study of the national prevalence, risk factors, and management of pneumonia in preschool children in China.

In the past decades, there has been a decreasing trend in the prevalence and mortality of pneumonia in preschool children due to the development of healthcare in China and the increased awareness of residents' medical care. According to the data of the China’s Under 5 Child Mortality Surveillance System (U5CMSS) database, the overall pneumonia of children under 5 years was reduced by 85.5% from 1996 to 2013 and the overall proportion of pneumonia deaths to total deaths was also declined from 23.4% in 1996 to 12.8% in 2013 in China [4].

In addition to the high mortality rate, pneumonia is also precipitating factor of other respiratory diseases, such as bronchial asthma [5–8]. Furthermore, some researches showed that there were a certain association between bronchial asthma and allergic rhinitis, this association is closely related to the overlap of susceptibility genes [9–11] and the common environmental risk factors [12, 13]. Studies have confirmed that allergic rhinitis and pneumonia among children have the same environmental risk factors [14].

In 2010, we established a research group China, Children, Homes, Health (CCHH) in 10 major cities in China. A total of 48,219 preschool children aged 2–8 years old were collected, and 25.5%–41.7% of preschool children had at least one episode of pneumonia [15]. CCHH’s research found that boys, low birthweight, duration of breastfeeding < 6 months, family allergy history and parental smoking were highly correlated with the lifetime incidence rate of children's pneumonia, and spotlight indoor environmental such as indoor humidity, cooking fuel, floor and wall materials were risk factors for pneumonia in children, good habits in daily life such as frequent drying of bedding in sunny conditions and increasing the cleaning frequency of children's rooms may be effective measures to prevent children's pneumonia [16]. Another CCHH’s study reported that PM2.5 and its some of chemical constituents in the lifetime annual average environment can increase the risk of pneumonia in children [17, 18].

In 2019, CCHH once again conducted a large, nationally representative sample of Chinese preschool children aged 2–8 years old to estimate the variation trend of prevalence of pneumonia. Additionally, we explored risk factors of pneumonia and association with other respiratory diseases, in order to provide important information needed for the development of national policies and programmers to further reduce the risk of pneumonia among children in China and other developing Asia–Pacific countries.

## Methods

### Study design and participants

CCHH was a large cross-sectional study that enrolled a representative sample of 63,663 and 52,812 Chinese preschool children in kindergartens in 2011 and 2019. In 2011, we used a multistage stratified cluster-sampling procedure, which considered geographical region, degree of urbanisation, economic development status, air pollution, and the gender and age distribution. In 2011, we first selected capital cities of ten provinces, autonomous regions, and municipalities, including Beijing, Shanghai, Nanjing, Chongqing, Wuhan, Taiyuan, Xian, Changsha, Haerbin and Urumqi. Secondary, we randomly selected 2–6 districts from each city. Thirdly, we randomly selected several kindergartens in each district of cities. Finally, we selected all the children in these kindergartens [15]. In 2019, method was used same as in 2011, 7 cities of Shanghai, Nanjing, Chongqing, Wuhan, Taiyuan, Changsha, and Urumqi were included. In this research, we select 7 cities surveyed jointly in 2011 and 2019, which including Shanghai, Nanjing, Chongqing, Wuhan, Taiyuan, Changsha, and Urumqi. The children whose basic information in the questionnaire is incomplete, non-residents (non local long-term residents since the mother's pregnancy date, or people who have moved across cities during this period), or more than 8 years old and less than 2 years old were excluded. Two studies in 2011 and 2019 all protocol was approved by the ethics review committees of the Fudan University (Shanghai, China). Written informed consent was obtained from the parents of all children that were enrolled into the study.

### Procedures

We defined pneumonia among children on the basis of a parents-reported history diagnosis at least one time by a physician in past time, using a pneumonia, asthma and other respiratory diseases and allergic diseases questionnaire from the International Study of Asthma and Allergies in Childhood (ISAAC) [19]. Questionnaire from the Swedish study “Dampness in Building and health (DBH)” about the home environments [20]. And according to the actual research needs, we modified some questions in the two parts of the questionnaire. The questionnaire was tested in a pilot study in 2010, and thereafter adjusted to improve readability [15]. For the details about questionnaire design, please refer to articles in other CCHH report [13–15, 21–28].

Uniform professional training for the responsible teachers involved in the survey class was conducted before the survey. A questionnaire was uniformly distributed to the parents of the children by the preschool teachers in their kindergartens, and explained questions from parents about questionnaire. Questionnaires were taken home by the child’s parent or other guardian and asked to be completed within 1 week, it was then handed to the responsible teacher at the kindergarten, who returned them uniformly to the local education bureau. Finally, the head of the subject group at each urban area retrieved the questionnaire.

Data on demographic, medical history (include pneumonia, asthma, allergy rhinitis, wheezing, et al.), duration of breast feeding, parental history of respiratory and allergy disease, parental smoking status, frequency of clean children's rooms (often was defined as clean at least twice a week, sometimes was defined as cleaning less than twice a week and more than once a week, rarely was defined as cleaning less than once every 2 weeks), frequency of dry the bedding in sunny conditions (often was defined as airing more than once every 1 week, sometimes was defined as airing less than once every 1 week, never was defined as never airing bedding in the sunny), material’s type of cooking and heating, presence of damp in the home (obvious mildew spots or damp on clothes / bedding / wall surface, or water leakage / seepage / water damage on floor / wall / ceilinge), decoration and newly purchased furniture in children's home, status of antibiotic use, medical history among children were identified according to the modified ISAAC questionnaire [15, 19]. Selection bias was addressed with multistage stratified cluster sampling, and technicians trained kindergarten teachers and explained the contents of the questionnaire to parents through teachers to avoid information bias. Multivariate logistic stepwise regression analyses were used to screen co-variates and adjust for confounding factors.

### Statistical analysis

Demographic, and some environmental factors potentially associated with the pneumonia were prespecified, include gender, birthweight, duration of breastfeeding, family allergy history, parental smoking, indoor humidity, cooking fuel, frequency of clean children's rooms, frequency of dry the bedding in sunny conditions [14, 16–18]. Other factors such as resident type (include urban residents, rural residents and suburb residents), decoration in the residence after the birth of the child, purchase of new furniture in the residence after the birth of the child, heating mode in winter, keep hairy pets in the house, air-conditioning and air-cleaner’s used in the residence also considerate in this research. We also reported prevalence of asthma, allergic rhinitis and wheezing, after determining other potential influencing factors by analysis of simple correlation, and after adjusted these factors, we analyzed association between pneumonia and asthma, allergic rhinitis, wheezing to assess impact of pneumonia on other respiratory diseases in children. In the estimation of missing data, we used the mean to interpolate the continuous variables, and used mode to interpolate the categorical variables.

We assessed the significance of differences by Student’s t test for continuous variables and by the χ^2^ test for categorical variables. We used multivariable logistic regression analyses to screen co-variates and examine the association between risk factors and pneumonia. We have conducted multiple tests and corrections on *p*-values. All statistical analyses were adapted two-sided test of level of α = 0.05, *p* < 0.05 was considered statistically significant. We used R software (version 4.1.2) for all statistical analyses.

### Role of the funding source

The funder of this study are the major members in CCHH, who designed these studies in cooperation with other members of CCHH. The funder had a role in data collection, data analysis, data interpretation, and writing of the report. The funder cooperates with other members of CCHH to interpret the data. Funder authors collaborated with academic authors in the development of the manuscript.

## Results

**Fig. 1:**
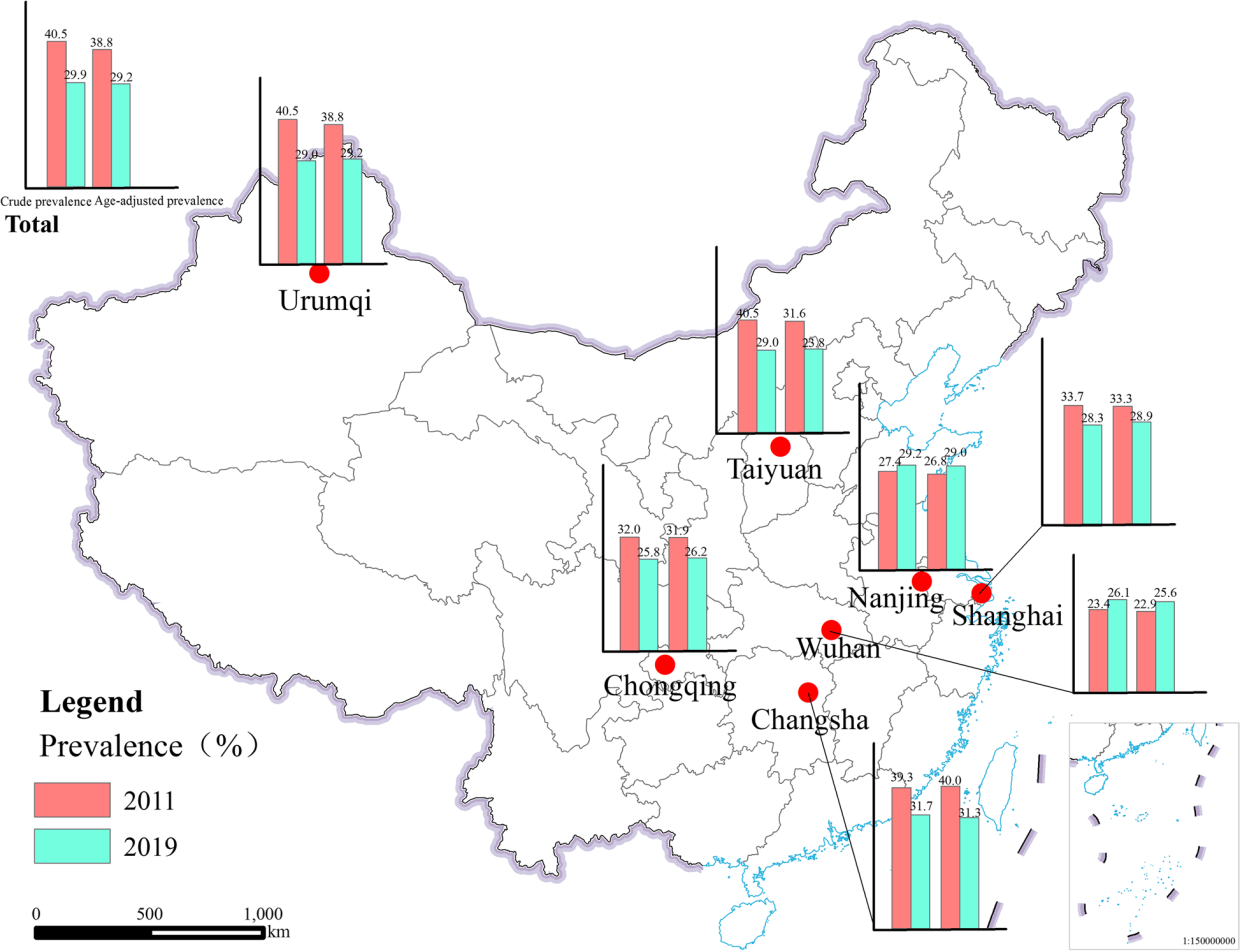
Prevalence of preschool children in seven cities of China in 2011 and 2019

In 2011, 63,663 preschool children were invited to participate in the survey; 15,444 refused to participate in the survey, and 10,402 children from Beijing, Xi'an, and Harbin were excluded because the above cities were not surveyed in 2019, and 6590 children were excluded because they had no information on pneumonia, gender, age, or younger than 2 years old or older than 8 years old, and non-permanent population. Finally, 31,277 preschool children (16,152 boys and 15,125 girls) were included in the study in 2011. In 2019, 52,812 preschool children were invited to participate in the survey; 6921 refused to participate in the survey, and 13,875 children were excluded because they had no information on pneumonia, gender, age, or younger than 2 years old or older than 8 years old, and non-permanent population. Finally, 32,016 preschool children (16,621 boys and 15,395 girls) were included in the study in 2019 (Additional file 1: Fig. S1). The distribution of pneumonia and non-pneumonia children by general characteristics and risk factors in 2011 and 2019 is summarized in Table 1. In 2011, 10,438 children with pneumonia were identified from the 31,277 participant, overall prevalence of pneumonia was 33.4%, age-adjusted prevalence of pneumonia was 32.7%. In 2019, 8905 children with pneumonia were identified from 32,016 participant, overall prevalence of pneumonia was 27.8%, age-adjusted prevalence of pneumonia was 26.4%. The overall prevalence and age-adjusted prevalence of pneumonia among preschool children in Chinese seven cities in 2011 and 2019 were shown in Fig. 1. In addition to Nanjing and Wuhan, the prevalence of pneumonia among children in other cities showed a decreasing trend, and the total prevalence of pneumonia also showed a decreasing trend. Pneumonia prevalence did not differ between age group in 2011 and 2019. In 2011 and 2019, children with pneumonia had significant differences in gender, urbanisation, duration of breastfeeding, birth weight, parental smoking, antibiotics use, history of parental asthma, history of parental allergy, frequency of putting bedding to sunshine, cooking fuel type, indoor dampness, home interior decoration, wall painting materials, flooring materials, indoor heating mode, indoor use air-conditioning, asthma, allergic rhinitis and wheezing (all *p* < 0.05). Premature birth, frequency of clean children's rooms, indoor keeping furry pets. In 2011, there was a significant difference in childhood pneumonia between home interior purchase new furniture, Indoor use air-conditioning (all *p* < 0.05), but there was no difference in 2019 (all *p* > 0.05) In 2019, there was a significant difference in childhood pneumonia between premature birth, frequency of clean children's rooms, indoor keeping furry pets (all *p* < 0.05), but there was no difference in 2011 (all *p* > 0.05). It is worth mentioning that the prevalence of pneumonia in children who used antibiotics in 2011 was higher than that of children who did not use antibiotics, but in 2019 was lower than 2011 (Additional file 2: Tab. S1).

**Table 1:** Demographics and risk factors by pneumonia in Chinese seven cities of preschool children population in 2011 and 2019

	2011		2019	
	No pneumonia	Pneumonia	No pneumonia	Pneumonia
Participants	20,839(66.6%)	10,438(33.4%)	23,111(72.2%)	8905(27.8%)
Boys	10,497(50.4%)	5655(54.2%)	11,767(50.9%)	4854(54.5%)
Girls	10,342(49.6%)	4783(45.8%)	11,344(49.1%)	4051(45.5%)
Mean age (years)	4.8 (1.3)	4.73(1.2)	4.4(1.1)	4.4(1.1)
Urban residents	15,546(74.6%)	8172(78.3%)	18,700(80.9%)	7169(80.5%)
Suburb residents	4461(21.4%)	2003(19.2%)	3581(15.5%)	1451(16.3%)
Rural residents	832(4.0%)	263(2.5%)	830(3.6%)	285(3.2%)
Duration of breastfeeding				
< 6 months	10,083(48.4%)	5670(54.3%)	8476(36.7%)	3585(40.3%)
≥ 6 months	10,756(51.6%)	4768(45.7%)	14,635(63.3%)	5320(59.7%)
Premature birth	837(4.0%)	454(4.3%)	417(1.8%)	235(2.6%)
Birth weight (g)	3382.1(479.8)	3366.9(484.7)	3336.2(510.2)	3311.5(512.6)
Parental smoking				
None	11,558(55.5%)	5565(53.3%)	16,885(73.1%)	5963(67.0%)
One	9163(44.0%)	4816 (46.1%)	6149(26.6%)	2901(32.6%)
Two	118(0.6%)	57(0.5%)	77(0.3%)	41(0.5%)
Used antibiotics	14,695(70.5%)	9219(88.3%)	7742(33.5%)	799(9.0%)
History of parental asthma				
None	20,311(97.5%)	10,033(96.1%)	22,674(98.1%)	8596(96.5%)
One	515(2.5%)	396(3.8%)	379(1.6%)	282(3.2%)
Two	13(0.1%)	9(0.1%)	58(0.3%)	27(0.3%)
History of parental allergy				
None	19,028(91.3%)	8955(85.8%)	16,888(73.1%)	5624(63.2%)
One	1656(7.9%)	1334(12.8%)	5277(22.8%)	2737(30.7%)
Two	155(0.7%)	149(1.4%)	946(4.1%)	544(6.1%)
Residence area				
< 75m^2^	8239(39.5%)	4015(38.5%)	5526(23.9%)	2092(23.5%)
≥ 75m^2^	12,600(60.5%)	6423(61.5%)	17,585(76.1%)	6813(76.5%)
Frequency of putting bedding to sunshine				
Never	625(3.0%)	375(3.6%)	635(2.7%)	200(2.2%)
Sometimes	6204(29.8%)	3534(33.9%)	7544(32.6%)	3389(38.1%)
Often	14,010(67.2%)	6529(62.6%)	14,932(64.6%)	5316(59.7%)
Frequency of clean children's rooms				
Rarely	299(1.4%)	135(1.3%)	572(2.5%)	247(2.8%)
Sometimes	3111(14.9%)	1727(16.5%)	4081(17.7%)	1806(20.3%)
Often	17,429(83.6%)	8576(82.2%)	18,458(79.9%)	6852(76.9%)
Cooking fuel type				
Natural gas/coal gas	15,667(75.2%)	8101(77.6%)	21,765(94.2%)	8479(95.2%)
Coal	1263(6.1%)	655(6.3%)	70(0.3%)	37(0.4%)
Electricity	2871(13.8%)	1125(10.8%)	1085(4.7%)	367(4.1%)
Wood	135(0.6%)	49(0.5%)	11(0.0%)	3(0.0%)
Other	903(4.3%)	508(4.9%)	180(0.8%)	19(0.2%)
Indoor dampness	2489(11.9%)	1658(15.9%)	3061(13.2%)	1548(17.4%)
Home interior decoration	3439 (16.5%)	2006 (19.2%)	2491 (10.8%)	897 (10.1%)
Home interior purchase new furniture	6977(33.5%)	3815(36.5%)	5022(21.7%)	1952(21.9%)
Wall painting materials				
Wall paper	2010(9.6%)	986(9.4%)	5800(25.1%)	2146(24.1%)
Emulsion paint	11,927(57.2%)	6268(60.0%)	9215(39.9%)	3794(42.6%)
Paint	2162(10.4%)	1228 (11.8%)	544(2.4%)	222(2.5%)
Wood	247(1.2%)	84 (0.8%)	171(0.7%)	33(0.4%)
Lime / cement	3293(15.8%)	1382 (13.2%)	533(2.3%)	171(1.9%)
Other	1200(5.8%)	490 (4.7%)	6848(29.6%)	2539(28.5%)
Flooring materials				
Solid wood / multi-layer solid wood	10,014(48.1%)	4941(47.3%)	9268(40.1%)	3448(38.7%)
Laminate / Composite wood	4333(20.8%)	2571(24.6%)	7084(30.7%)	3001(33.7%)
Bamboo	313(1.5%)	132(1.3%)	218(0.9%)	62(0.7%)
Ceramic tile / stone / cement	5550(26.6%)	2508(24.0%)	6020(26.0%)	2245(25.2%)
PVC / plastics / plastic leather	265(1.3%)	124(1.2%)	75(0.3%)	27(0.3%)
Other	364(1.7%)	162(1.6%)	446(1.9%)	122(1.4%)
Indoor heating mode				
No heating	2828(13.6%)	1223(11.7%)	6677(28.9%)	2552(28.7%)
Individual household heating	13,446(64.5%)	6691(64.1%)	10,391(45.0%)	4164(46.8%)
Central heating	4188(20.1%)	2357(22.6%)	5161(22.3%)	1890(21.2%)
Other	377(1.8%)	167(1.6%)	882(3.8%)	299(3.4%)
Indoor use air-conditioning	14,234(68.3%)	6916(66.3%)	18,555(80.3%)	7252(81.4%)
Indoor use air purifier	1045(5.0%)	595(5.7%)	8428(36.5%)	3267(36.7%)
Indoor keeping furry pets				
Yes	2469 (11.8%)	1164 (11.2%)	2625 (11.4%)	1099 (12.3%)
No	18,370 (88.2%)	9274 (88.8%)	20,486 (88.6%)	7806 (87.7%)
Asthma	943(4.5%)	1528(14.6%)	490(2.1%)	723(8.1%)
Allergic rhinitis	1614(7.7%)	1537(14.7%)	2117(9.2%)	1653(18.6%)
Wheezing	4337(20.8%)	3772(36.1%)	932(4.0%)	1290(14.5%)

Data are shown as number (%) or mean (SE). Pneumonia, asthma, allergic rhinitis were defined as physician-diagnosed since birth of children. Wheezing was defined as parents reported that child has great difficulty breathing or the respiratory muscles of the child are all involved in breathing, and the respiratory rate of the child is faster than normal. Premature birth was defined as delivery under 37 weeks of pregnancy. Used antibiotics was defined as child has been injected or taken since birth, such as but not limited to penicillin, azithromycin, cephalosporin, etc. Parental smoking was defined as parents smoked equal to or more than 100 cigarettes in the lifetime

Compared to 2011, proportion of breastfeeding duration > 6 months, children with neither parent smoking nor asthma, ratio of natural gas / gas in cooking materials, indoor dampness, renovation, and newly purchased furniture, percentage of wall material wallpapers and other wall materials, and indoor use of air conditioning and air cleaners were higher in 2019; The proportion of preterm infants, birth weight, children with neither parent allergic, percentage of children ever used antibiotics, and kids room cleaning frequency, Latex paint, paint, wood panels, cement / Stone ratios, ratio of indoor no heating, centralized heating, and other heating modes were lower in 2019; Often and never in sunlight is enough to dry bedding the share is lower, while sometime and rarely share is higher in 2019; The floor materials solid wood / multi layer solid wood and polyvinyl chloride (PVC) / plastics / plastic leader were lower and the laminate / composite wood was higher in 2019; The ratio of indoor no heating, centralized heating, and other heating modes was higher, while individual heating was lower in 2019 (all *p* < 0.05). The most important was prior antibiotic use in 2011, 23,914 (76.5%) of 31,277 children surveyed, and 8541 (36.7%) of 32,016 children surveyed in 2019 (Additional file 2: Tab. S1).

In 2011, 2471 of 31,277 children surveyed had asthma, total crude prevalence was 7.9%, 3151 had allergic rhinitis, total crude prevalence was 10.1%, 8109 were wheezing, and total crude prevalence was 25.9%. In 2019, 1213 of 32,016 children surveyed had asthma, total crude prevalence was 3.8%, 3770 children had allergic rhinitis, total crude prevalence was 11.8%, 2222 children had wheezing, total crude prevalence was 6.9%. The total crude prevalence of asthma, allergic rhinitis, and wheezing were lower in 2019 than in 2011 (Additional file 2: Tab. S1).

Additional file 1: Fig. S2 demonstrated the co-morbidities of wheezing, asthma, and allergic rhinitis with pneumonia in children in 2011 and 2019. In 2011, 1528 children developed pneumonia simultaneously in the context of having asthma, 1537 children developed pneumonia simultaneously in the context of having allergic rhinitis, and 3772 children developed simultaneously in the context of wheezing. In 2019, 724 children developed pneumonia simultaneously in the context of asthma, 1654 children developed pneumonia simultaneously in the context of allergic rhinitis, and 1291 children developed pneumonia simultaneously in the context of wheezing.

In 2011, the age-adjusted prevalence of pneumonia was 62.2% (95% CI 60.3–64.1) in children with asthma, 30.3% (95% CI 29.8–30.8) in non-asthma children, the age-adjusted prevalence of pneumonia was 47.6% (95% CI 45.9–49.4) in children with allergic rhinitis, 31.1% (95% CI 30.6–31.6%) in children with non-allergic rhinitis, the age-adjusted prevalence of pneumonia was 47.1%(95% CI 46.0–48.2) in wheezing children, 28.4% (95% CI 27.8–29.0) in non-wheezing children. In 2019, the age-adjusted prevalence of pneumonia was 59.5% (95% CI 56.6–62.2) in children with asthma, 26.5% (95% CI 26.0–27.0) in non-asthma children, the age-adjusted prevalence of pneumonia was 44.1% (95% CI 42.5–45.7) in children with allergic rhinitis, 25.6% (95% CI 25.1–26.2) in children with non-allergic rhinitis, the age-adjusted prevalence of pneumonia was 57.3% (95% CI 55.2–59.4) in wheezing children, 25.6% (95% CI 25.1–26.1) in non-wheezing children. The prevalence of pneumonia in 2011 was higher in children with asthma, allergic rhinitis, and wheezing than 2019 compared to unaffected children (all *p* < 0.0001, Additional file 2: Tab. S1).

By multivariate logistic stepwise regression analysis, in 2011, gender, age, urbanisation, duration of breastfeeding, birth weight, parental smoking, used antibiotics, history of parental allergy, frequency of putting bedding to sunshine, cooking fuel type, indoor dampness, home interior decoration, wall painting materials, flooring materials, indoor heating mode, indoor use air-conditioning, asthma, allergic rhinitis, and wheezing were eventually incorporated into multivariate adjusted analyses. In 2019, gender, urbanisation, duration of breastfeeding, birth weight, parental smoking, used antibiotics, history of parental asthma, history of parental allergy, cooking fuel type, indoor use air-conditioning, asthma, allergic rhinitis, and wheezing were eventually incorporated into multivariate adjusted analyses. Of these, girls, duration of breastfeeding ≥ 6 months, and indoor use of air-conditioning were all associated with a reduced risk of pneumonia; Smoking by one or two of the parents, history of parental allergy, and indoor dampness, asthma, allergic rhinitis, and wheezing are all associated with an increased risk of pneumonia in both 2011 and 2019 (Table 2).

**Table 2:** Multiple adjusted odds ratios of pneumonia in the Chinese seven cities of preschool children in 2011 and 2019

	2011		2019	
	OR(95%CI)	*p* value	OR(95%CI)	*p* value
Girls	0.91(0.87–0.96)	0.0002	0.92(0.87–0.97)	0.0019
Age			.	.
2–4	1.00		.	.
4–6	1.11(1.03–1.20)	0.0052	.	.
6–8	0.98(0.90–1.07)	0.6518	.	.
Urbanisation				
Urban	1.00		1.00	
Suburb	0.96(0.90–1.02)	0.1681	1.10(1.02–1.18)	0.0093
Rural	0.85(0.73–0.99)	0.0387	1.14(0.99–1.32)	0.0744
Duration of breastfeeding ≥ 6 months	0.83(0.79–0.88)	< 0.0001	0.92(0.87–0.97)	0.0031
Premature birth	.	.	1.29(1.08–1.55)	0.0051
Birth weight (g)				
< 2500	1.11(0.98–1.25)	0.0997	1.17(1.02–1.35)	0.0284
2500 ~ 4000	1.00		1.00	
≥ 4000	0.88(0.80–0.97)	0.0125	0.97(0.91–1.04)	0.4496
Parental smoking				
None	1.00		1.00	
One	1.12(1.07–1.18)	< 0.0001	1.30(1.23–1.38)	< 0.0001
Two	1.02(0.73–1.42)	0.8983	1.42(0.94–2.12)	0.0924
Used antibiotics	2.71(2.52–2.90)	< 0.0001	0.22(0.21–0.24)	< 0.0001
History of parental asthma	.	.		
None	.	.	1.00	
One	.	.	1.23(1.03–1.46)	0.0202
Two	.	.	0.96(0.59–1.54)	0.8764
History of parental allergy				
None	1.00		1.00	
One	1.21(1.12–1.32)	< 0.0001	1.20(1.13–1.27)	< 0.0001
Two	1.33(1.04–1.69)	0.0203	1.22(1.08–1.37)	0.0014
Frequency of putting bedding to sunshine			.	.
Never	1.00		.	.
Sometimes	0.92(0.80–1.06)	0.2412	.	.
Often	0.82(0.71–0.94)	0.0049	.	.
Cooking fuel type				
Natural gas/coal gas	1.00		1.00	
Coal	1.01(0.91–1.12)	0.8835	1.58(1.02–2.52)	0.0356
Electricity	0.87(0.80–0.94)	0.0005	0.96(0.84–1.09)	0.5246
Wood	0.82(0.57–1.15)	0.2558	0.52(0.10–2.06)	0.3914
Other	1.12(0.99–1.26)	0.0670	0.40(0.23–0.63)	0.0003
Indoor dampness	1.24(1.15–1.33)	< 0.0001	1.16(1.08–1.24)	< 0.0001
Home interior decoration	1.11(1.04–1.19)	0.0013	.	.
Wall painting materials			.	.
Wall paper	1.00		.	.
Emulsion paint	1.06(0.98–1.16)	0.1623	.	.
Paint	1.16(1.04–1.29)	0.0084	.	.
Wood	0.83(0.63–1.09)	0.1975	.	.
Lime / cement	0.99(0.88–1.10)	0.8458	.	.
Other	0.94(0.82–1.08)	0.4157	.	.
Flooring materials			.	.
Solid wood / multi-layer solid wood	1.00		.	.
Laminate / Composite wood	1.08(1.02–1.16)	0.0126	.	.
Bamboo	0.81(0.65–1.00)	0.0556	.	.
Ceramic tile / stone / cement	1.01(0.94–1.08)	0.8476	.	.
PVC / plastics / plastic leather	0.97(0.77–1.21)	0.7777	.	.
Other	1.09(0.89–1.33)	0.4046	.	.
Indoor heating mode			.	.
No heating	1.00		.	.
Individual household heating	1.04(0.96–1.13)	0.2924	.	.
Central heating	1.18(1.07–1.30)	0.0090	.	.
Other	1.05(0.85–1.29)	0.6309	.	.
Indoor use air-conditioning	0.85(0.80–0.90)	< 0.0001	0.89(0.83–0.95)	0.0009
Asthma	2.38(2.17–2.61)	< 0.0001	1.88(1.64–2.15)	< 0.0001
Allergic rhinitis	1.36(1.25–1.47)	< 0.0001	1.57(1.45–1.69)	< 0.0001
Wheezing	1.64(1.55–1.74)	< 0.0001	2.43(2.20–2.68)	< 0.0001

After a multivariate logistic stepwise regression analysis, the co-variates that were finally included in the analysis are listed in the table. ORs of 1.00 indicate reference values. Pneumonia, asthma, allergic rhinitis were defined as physician-diagnosed since birth of children. Wheezing was defined as parents reported that the child has great difficulty breathing or the respiratory muscles of the child are all involved in breathing, and the respiratory rate of the child is faster than normal since birth. Premature birth was defined as delivery under 37 weeks of pregnancy. Used antibiotics was defined as child has been injected or taken since birth, such as but not limited to penicillin, azithromycin, cephalosporin, etc. Parental smoking was defined as parents smoked equal to or more than 100 cigarettes in the lifetime. OR = odds ratio. ^*^p value were OR < 0.0001 maybe due to too few subjects for these co-variates

In 2011, age 4–6 years, rurality, birth weight ≥ 4000 g, frequency of pumping bedding to sunshine (often), cooking fuel type (electronic) were all associated with a reduced risk of pneumonia, used antibodies, home interior decortication, wall coating materials (paint), flowing materials (laminate / composite wood), Indoor heating mode (central heating) are all associated with an elevated risk of pneumonia. These relationships were not represented in 2019. In 2019, use of antibiotics, cooking fuel type (other), have all been associated with a reduced risk of pneumonia, urbanisation (suburb), birth weight < 2500 g, history of parental asthma, and cooking fuel type (coal) have all been associated with an elevated risk of pneumonia (Table 2).

In 2011, considering only children with duration of breastfeeding ≥ 6 months, urbanisation (rural), birth weight ≥ 4000 g, home interior degradation, wall coating materials (paint), floating materials (laminate / composite wood) were no longer associated with the prevalence of pneumonia in children, whereas age 2–4 years was associated with an elevated risk of pneumonia; Considering only children who had never used antibiotics, urbanisation (rural), birth weight ≥ 4000 g, cooking fuel type (Electronic), home interior decortication, wall coating materials (paint), floating materials (laminate / composite wood), indoor healing mode (central healing) were not associated with the prevalence of pneumonia; Considering only children who had ever used antibiotics, two in history of parental allergy were no longer associated with childhood pneumonia, and floating materials (bamboo) were associated with a reduced risk of pneumonia, 2–4 years of age, birth weight < 2500 g, and cooking fuel type (other) were associated with an elevated risk of pneumonia (Additional file 2: Tab. S2).

In 2019, considering only children with duration of breastfeeding ≥ 6 months, birth weight < 2500 g, history of parental asthma (one person) was no longer associated with childhood pneumonia; Considering only children who had not used antibiotics, the cooking fuel type (coal) was no longer associated with pneumonia in children; Among children considered for antibiotic use only, gender, urbanisation (suburb), duration of breastfeeding ≥ 6 months, premature birth, birth weight < 2500 g, history of parental asthma (one person), history of parental allergy (one person), indoor dampness, and indoor use air conditioning were no longer associated with childhood pneumonia (Additional file 2: Tab. S2).

We analyzed the effect of pneumonia on asthma, allergic rhinitis, and wheezing after setting the outcome variables to asthma, allergic rhinitis, and wheezing, respectively, using the same multivariate logistic stepwise regression. After multivariable adjustment, pneumonia was associated with an elevated risk of childhood asthma, allergic rhinitis, and wheezing in both 2011 and 2019 (Additional file 2: Tab. S3).

## Discussion

Our results from a large comprehensive preschool pneumonia survey in seven cities in China showed that the age-adjusted prevalence of pneumonia among children was 32.7% in 2011 and 26.4% in 2019, which is lower than that in 2011, and according to the report that the proportion of children dying from pneumonia in China under 5 years of age also decreased [4], which is a good trend. But since pneumonia in addition to causing death in children, pneumonia was also a predisposing factor for other respiratory diseases according to previous reports [5–8]. This survey similarly found that pneumonia in children was a significant factor for increased asthma, allergic rhinitis, and wheezing. These diseases can have a significant impact on child health, and in particular, the risk of wheezing attributable to pediatric pneumonia was higher in 2019 (OR = 2.49, 95% CI 2.25–2.75) compared with 2011 (OR = 1.64, 95% CI 1.55–1.74). The risk of allergic rhinitis among children with pneumonia was also higher in 2019 (OR = 1.60, 95% CI 1.48–1.73) compared with 2011 (OR = 1.41, 95% CI 1.29–1.53) (Additional file 2: Tab. S3). In addition, there is also no strict surveillance agency for pneumonia in children in China, so pneumonia in children remains an important public health challenge. Meanwhile, there are still many low and middle-income countries (LMICs) in the world, who still face high pneumonia prevalence in children, high pneumonia lethality, and are still rising year by year [1, 29–31]. Therefore, as the largest developing country in the world, our study is not only able to generate a positive response for preschool child health promotion in China, but also as the largest developing country in the world. Our research experience can provide a strong basis for prevention and control of pneumonia in children in those LMICs.

To our knowledge, CCHH is the largest research team focused on common diseases in preschool children in China. The CCHH studies all used a strict sampling design and used questionnaires that have been validated and revised several times [19, 20]. Our present study is also the only cross-sectional survey of interrupted time series deployed for pneumonia in Chinese children. We put forward more persuasive policy on the management of pneumonia in preschool children by contrasting the prevalence and influencing factors of pneumonia in 2011 and 2019, and comparing the proportion of these factors among children overall in the two years, and the combined analysis leads to the reason that the prevalence of pneumonia in 2019 is lower than that in 2011.

Based on the results of this study, we identified several contributing factors associated with pneumonia among preschool children in China in 2011 vs 2019. First, the prevalence of pneumonia was lower in girls than boys, which was confirmed by CCHH in previous studies of pediatric pneumonia in different cities [14, 25]. This may result from boys being more active and more often exposed to risk factors that cause pneumonia than girls. Second, breastfeeding duration ≥ 6 months was a significant protective factor against childhood pneumonia. There are many studies proposing that breastfeeding in developing countries is the most cost-effective health intervention for reducing pneumonia related deaths in infancy [2, 32, 33]. Therefore, strengthening health education about breastfeeding is an effective measure to control pneumonia in children. Third, the use of air conditioning at home is one of the factors that contribute to the reduced risk of pneumonia in children, the reasons for which in our analysis may be related to the ability of air conditioning to effectively reduce indoor humidity. The CCHH in earlier study found that signs of indoor moldiness or characterization of dampness was associated with almost all respiratory and allergic diseases in children [13], while this study similarly found that the phenomenon of indoor dampness in 2011 vs 2019 was a contributing factor to the increasing risk of pneumonia in children. Therefore, using air conditioning indoors and reducing indoor humidity may be measures to prevent pneumonia in children. Fourth, smoking in one of the parents is one of the major factors contributing to the increasing risk of pneumonia in children. Because of the small number of maternal smokers in both surveys, no association between smoking in both parents and pneumonia in children was found. Parental smoking is well known to have adverse effects on child health, one of which arises from the effects of smoking on the reproductive system of parents, leading to adverse effects on the growth and development of the fetus in the mother [34, 35]. On the other hand, parental smoking, especially paternal smoking, not only increases the risk of passive smoking in children, but also increases the risk of maternal passive smoking during pregnancy, and thereby affects the child's lung development during the fetal period and childhood growth and development, leading to impaired early lung function [36–38]. Therefore, advocacy education on the hazards of tobacco should be enhanced to reduce the damage caused by parental smoking on child health. Finally, parental exposure to allergic diseases is a major contributor to the elevated risk of pneumonia in children. Many studies have previously reported a high heritability of allergic diseases, for example a parent or mother history of allergic diseases was significantly associated with the development of eczema and allergic rhinitis in childhood [9, 39, 40]. According to the present survey, children with allergic rhinitis were found to have a higher risk of pneumonia (OR = 1.36, 95% CI 1.25–1.47), which may be one of the reasons why parents with allergic diseases have an elevated risk of pneumonia in children.

In addition to the same influencing factors in 2011 vs 2019, there are some differences in influencing factors of pneumonia in children in 2011 vs 2019. First, children in rural areas had a lower pneumonia prevalence risk in 2011, while those in suburban areas had a lower pneumonia prevalence risk in 2019, which may be due to the increasing urbanization rate in China. Preterm infants have a higher risk of pneumonia in 2019, which is not reflected in 2011. Prematurity poses significant hazards that can affect fetal growth and development, including a significant association between bronchopulmonary dysplasia and prematurity reported by Northway in 1961 [41]. Meanwhile, preterm infants are prone to low birth weight, and low birth weight may cause growth retardation in children and increase the risk of multiple diseases after day [42]. 2019 also similarly found that low birth weight children have a higher risk of pneumonia. There are some other factors that differ between 2011 and 2019. Frequent exposure to soiled bedding in 2011 was a childhood pneumonia protective factor, whereas no association was detected in 2019. Children who cooked fuel types with electricity relative to natural gas / gas in 2011 had lower pneumonia prevalence risk, while children who cooked with other material relative to natural gas / gas in 2019 had lower pneumonia prevalence risk. Both wall material and floor material were associated with the prevalence of childhood pneumonia in 2011, whereas these associations were not found in 2019. Children whose parents have asthma in 2019 have a higher risk of pneumonia. In addition to the above factors, the largest difference in the influencing factors of pneumonia between 2011 and 2019 children is the use of antibiotics. Children who had used antibiotics in 2011 had a higher risk of pneumonia (OR = 2.71, 95% CI 2.52–2.90), while children who had used antibiotics in 2019 had a lower risk of pneumonia (OR = 0.22, 95% CI 0.21–0.24).

Comprehensive analysis of the reasons for the decline in pneumonia in children in 2019 compared to 2011, which is the most associated with the following points. First breastfeeding for ≥ 6 months is one of the important factors, and our country's work on the health education of breastfeeding is highly valued, and related work has been carried out in many forms [43]. This is visible from the 49.6% percentage of the overall pediatric breastfeeding duration ≥ 6 months in 2011 to the 62.3% increase in 2019 in this study (Additional file 2: Tab. S4). Second, the standardized use of antibiotics may be the most important reason for the lower prevalence of pneumonia among children in 2019 compared with 2011. The present investigation found, by multivariate analysis, that antibiotic use was a contributing factor to the increasing risk of childhood pneumonia in 2011. A study based on a random sample of 6 provincial primary care facilities in 2011 showed that 25% of outpatient prescriptions and 68% of inpatient prescriptions had problems with antibiotic use [44, 45]. An immediate consequence of antibiotic misuse is the development of resistance to antibiotics, which can directly cause 1.27 million deaths annually [46]. China has introduced a series of policies on the standardized use of clinical antibiotics and the spread of knowledge on the rational application of antibiotics since 2010 to now [47–52], and has achieved great success. This is visible from 76.5% of surveyed children's antibiotic use in 2011 to a decline to 26.7% in 2019 (Additional file 2: Tab. S5). And multivariate analysis results the use of antibiotics in 2019 children is a protective factor for pneumonia in children (Additional file 2: Tab. S2). This may be explained by the reasonable use of antibiotics when children develop bacterial infections of the respiratory tract in early life, allowing disease progression to be controlled. Third, the proportion of individuals with at least one parent who smoked was 45.3% in 2011, which decreased to 28.7% in 2019. Fourth, indoor renovation materials contain a large number of volatile organic compounds, among which formaldehyde, benzene, toluene, xylene are the most common several substances [53]. Children, after inhalation of such substances at high concentrations, irritate the respiratory tract, inducing respiratory disease. While formaldehyde is the most representative substance, the current study [54] confirmed that formaldehyde can cause respiratory injury. Not only that, substances such as formaldehyde can also cross the placental barrier and act on the embryo, causing abnormal fetal growth and development. Changes in indoor renovation materials are an important cause of the decreasing prevalence of pneumonia in children. There are differences in the composition of flooring materials and wall materials in 2011 and 2019, and several national standards have been established in China after 2011 on the limits of hazardous substances from indoor decoration renovation materials [55–59], which makes the content of hazardous substances in renovation materials effectively controlled. In addition, outdoor environmental pollution, which was not addressed in this study, has long been shown by CCHH to be associated with the prevalence of pneumonia in children [17, 18]. China has achieved remarkable results on air pollution governance in recent years, and air pollutants such as PM2.5 all show a decreasing trend year by year, which may also be the reason why the prevalence of pneumonia among children is decreasing in 2019.

Our study still has the following limitations. First, our study was only conducted in 7 capital cities throughout the country, and the sample could not be representative of the whole country. Follow up sampling surveys optimize the mode of sampling to cover as many different territories as possible. Second, the onset of pneumonia is time-consuming, and there may be uniform children with multiple episodes of pneumonia, which would make the prevalence of pneumonia underestimated. There were a subset of parents of children who refused to participate in this research investigation, thus it may have some influence on the findings. Then, we currently have difficulty with judging whether the duration of antibiotic use preceded the onset of pneumonia in children, so this is one of the reasons why we stratified our analyses by antibiotic use. But this also cannot fully address the bias introduced by this limitation, and we will follow up with further studies focusing on the association between antibiotic use and pneumonia in children. Finally because of the poor power of causal arguments in cross-sectional studies, follow-up is required to make it clear whether the factors found to be associated with pneumonia in this study are causally linked to pneumonia through a population-based cohort study.

To our knowledge, currently China has only issued two guidelines for the management of community-acquired pneumonia in children in 2007 [60, 61] and 2013 [62, 63]. Guidelines for Diagnosis and Treatment Community-acquired Pneumonia in Children was issued in 2019 in China [64]. The monitoring and management system for pneumonia in children is currently lacking still missing. For this, we propose that a well-established surveillance system should be established in the community, not only for pneumonia in children, but also for other common diseases in children.

## Conclusions

Our findings call for efforts to improve the prevention of pneumonia among preschool children in China. Enhancing maternal and child health preaching, raising awareness of the importance of breastfeeding, standardizing antibiotic use, and controlling exposure to indoor environmental risk factors are important public health priorities to reduce the burden of pneumonia in Chinese children.

## Supplementary Information

**Additional file 1: Fig. S1.** Flowchart of this study. **Fig. S2.** Cross-prevalence of pneumonia, asthma, allergic rhinitis and wheezing among preschool children in the Chinese seven cities in 2011 and 2019.

**Additional file 2:** **Tab.S1.** Age-specific and age-adjusted prevalence of pneumonia among preschool children in the Chinese seven cities in 2011 and 2019 by gender. **Tab.S2.** Multiple adjusted odds ratios of pneumonia in the Chinese seven cities of preschool children in 2011 and 2019 by duration of breastfeeding and antibiotics use. **Tab. S3.** Multiple adjusted odds ratios of asthma, allergic rhinitis and wheezing and in the Chinese seven cities of preschool children in 2011 and 2019. **Tab.S4.** Age-specific and age-adjusted prevalence of pneumonia among preschool children in the Chinese seven cities in 2011 and 2019 by duration of breastfeeding. **Tab. S5.** Age-specific and age-adjusted prevalence of pneumonia among preschool children in the Chinese seven cities in 2011 and 2019 by antibiotics use.

## Data Availability

The datasets generated during and/or analysed during the current study are available from the corresponding author on reasonable request.

## Abbreviations

AR: Allergic rhinitis
CCHH: China, Children, Homes, Health
CI: Confidence interval
DBH: Dampness in Building and health
ISAAC: International Study of Asthma and Allergies in Childhood
LMICs: Low and middle-income countries
OR: Odds ratio
PM2.5: Fine particulate matter
PVC: Polyvinyl chloride
U5CMSS: China’s Under 5 Child Mortality Surveillance System
