# Supplementary material for: Prevalence, risk factors, impact and management of pneumonia among preschool children in Chinese seven cities: a cross-sectional study with interrupted time series analysis
Source: BMC Med. 2023 Jun 26;21:227. doi: 10.1186/s12916-023-02951-2 (PMC10294363; doi:10.1186/s12916-023-02951-2)
Supplement: Supplementary file 1 — Additional file 1: Fig. S1. Flowchart of this study. Fig. S2. Cross-prevalence of pneumonia, asthma, allergic rhinitis and wheezing among preschool children in the Chinese seven cities in 2011 and 2019. [file 12916_2023_2951_MOESM1_ESM.pdf]

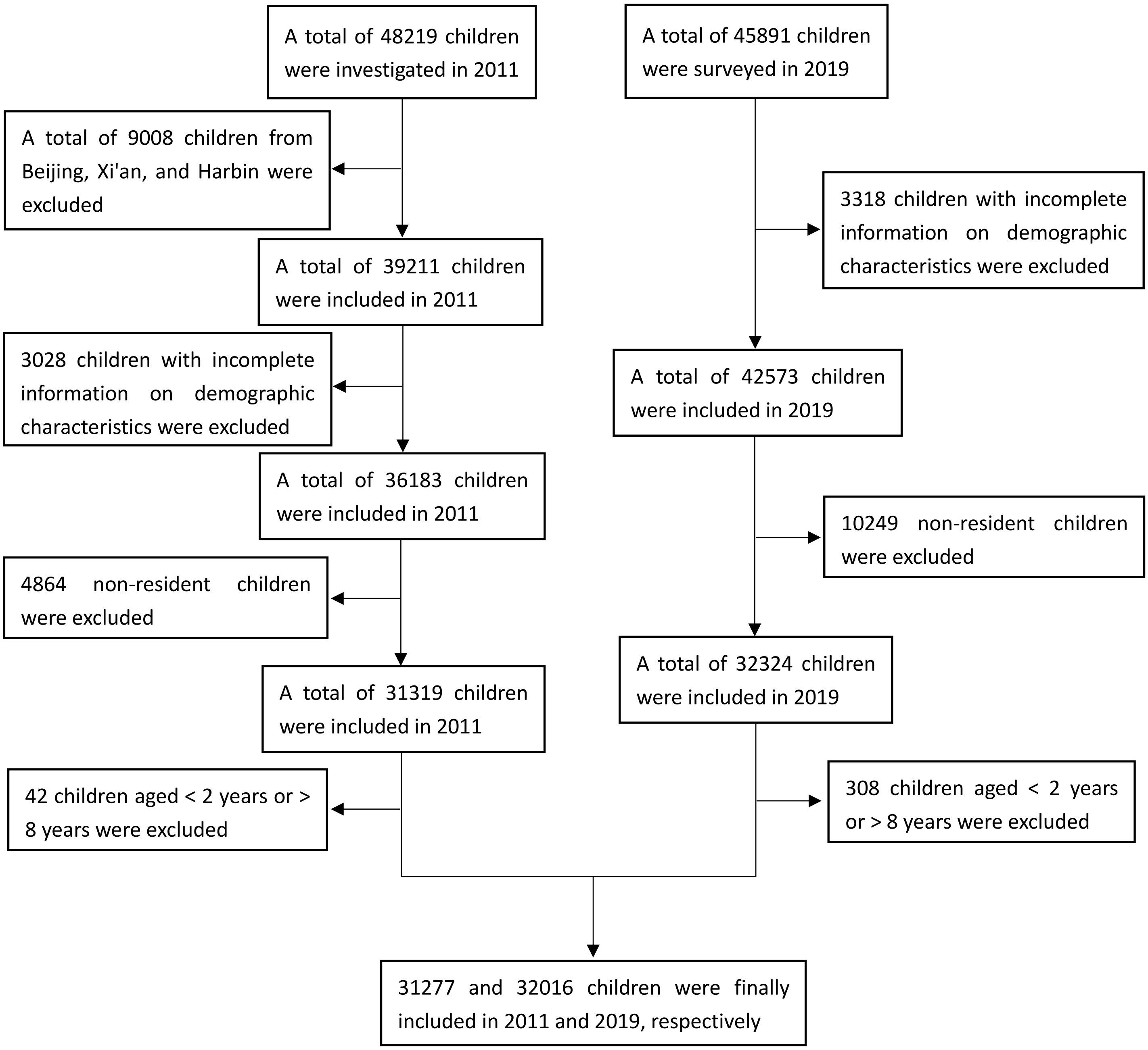

Figure S1: Flowchart of this study.

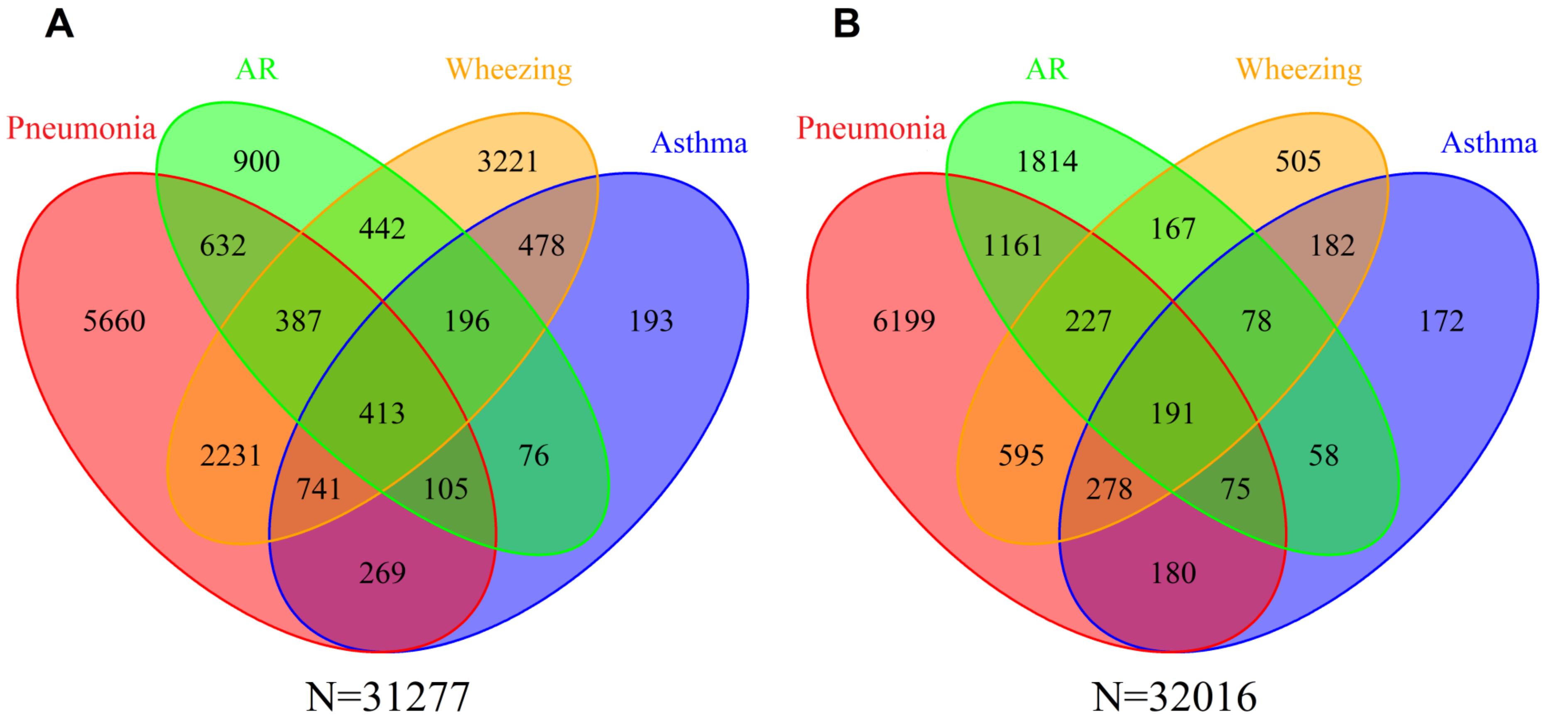

Figure S2: Cross-prevalence of pneumonia, asthma, allergic rhinitis and wheezing among preschool children in the Chinese seven cities in 2011 and 2019.
