# Supplementary material for: Prevalence, risk factors, impact and management of pneumonia among preschool children in Chinese seven cities: a cross-sectional study with interrupted time series analysis
Source: BMC Med. 2023 Jun 26;21:227. doi: 10.1186/s12916-023-02951-2 (PMC10294363; doi:10.1186/s12916-023-02951-2)
Supplement: Supplementary file 2 — Additional file 2: Tab.S1. Age-specific and age-adjusted prevalence of pneumonia among preschool children in the Chinese seven cities in 2011 and 2019 by gender. Tab.S2. Multiple adjusted odds ratios of pneumonia in the Chinese seven cities of preschool children in 2011 and 2019 by duration of breastfeeding and antibiotics use. Tab. S3. Multiple adjusted odds ratios of asthma, allergic rhinitis and wheezing and in the Chinese seven cities of preschool children in 2011 and 2019. Tab.S4. Age-specific and age-adjusted prevalence of pneumonia among preschool children in the Chinese seven cities in 2011 and 2019 by duration of breastfeeding. Tab. S5. Age-specific and age-adjusted prevalence of pneumonia among preschool children in the Chinese seven cities in 2011 and 2019 by antibiotics use. [file 12916_2023_2951_MOESM2_ESM.pdf]

Supplementary Text

Performance of the ISAAC and DBH screening questionnaire items in investigate influencing factors of respiratory and allergic diseases

International Study of Asthma and Allergies in Childhood (ISAAC)[1] and Dampness in Building and health (DBH)[2] screening questionnaire have been verified by relevant research institutes,[3-5] and have been translated into Chinese and used in research group China, Children, Homes, Health (CCHH), and we conducted a follow-up survey on 300 respondents in Chongqing and collected 206 copies. The short questionnaire contains five questions, including home address, gender, wheezing in the past 12 months, whether there are visible wet spots at home and family smoking. The results showed that there was no significant difference between the short questionnaire participants and the long questionnaire participants (Table).[6] Of course, this survey has unavoidable limitations of all questionnaires. The collected data analyzed depends on the parents' retrospective reports, which may contain bias caused by memory bias. For discussion of some biases, please refer to the research results of each city.[7-16]

Table: Comparison of long questionnaire and short questionnaire in Chongqing in 2011[6]

	short questionnaire	long questionnaire	p value
Sample size	206	5299	
Questionnaire recovery rate	68.7	74.5	
Residential location			0.070
Rural	11.2	10.4	
Suburb	24.7	18.7	
Urban	54.1	70.9	
Gender			0.478
Boys	53.9	51.3	
Gilrs	46.1	48.7	
Wheezing in the past 12 months	26.3	20.6	0.054
Visible wet spots at home	10.8	8.1	0.181
Members of the family smoke	71.4	65.6	0.099

[1] Asher M, Keil U, Anderson H, et al. International Study of Asthma and Allergies in Childhood (ISAAC): Rationale and methods. Eur Respir J. 1995;8:483-91.

[2] Bornehag CG, Sundell J, Sigsgaard T. Dampness in buildings and health (DBH): Report from an ongoing epidemiological investigation on the association between indoor environmental factors and health effects among children in Sweden. Indoor Air. 2004;14:59-66.

[3] Chan HH, Pei A, Van Krevel C, et al. Validation of the Chinese translated version of ISAAC core questions for atopic eczema. Clin Exp Allergy. 2001;31:903–7.

[4] Sun YX, Sundell J, Zhang YF. Validity of building characteristics and dorm dampness obtained in a self-administrated questionnaire. Sci Total Environ. 2007;387:276–82 .

[5] Engman LH, Bornehag CG, Sundell J. How valid are parents' questionnaire responses regarding building characteristics, mouldy odour, and signs of moisture problems in Swedish homes?. Scand J Public Health. 2007;35:125–32.

[6] Zhang YP, Li BZ, Huang C, et al. Ten cities cross-sectional questionnaire survey of children asthma and other allergies in China. Chinese Sci Bull. 2013;58:4182-9.

[7] Zhao ZH, Zhang X, Liu RR, et al. Prenatal and early life home environment exposure in relation to preschool children's asthma, allergic rhinitis and eczema in Taiyuan, China. Chinese Sci Bull. 2013;58:4245-51.

[8] Wang TT, Zhao ZH, Yao H, et al. Housing characteristics and indoor environment in relation to children's asthma, allergic diseases and pneumonia in Urumqi, China. Chinese Sci Bull. 2013;58:4237-44.

[9] Huang C, Hu Y, Liu W, et al. Pet-keeping and its impact on asthma and allergies among preschool children in Shanghai, China. Chinese Sci Bull. 2013;58:4203-10.

[10] Liu W, Huang C, Hu Y, et al. Associations between indoor environmental smoke and respiratory symptoms among preschool children in Shanghai, China.Chinese Sci Bull. 2013;58:4211-6.

[11] Zhang M, Wu Y, Yuan Y, et al. Effects of home environment and lifestyles on prevalence of atopic eczema among children in Wuhan area of China. Chinese Sci Bull. 2013;58:4217-22.

[12] Zhang M, Zhou ES, Ye X, et al. Indoor environmental quality and the prevalence of childhood asthma and rhinitis in Wuhan area of China. Chinese Sci Bull. 2013;58:4223-9.

[13] Zheng XH, Qian H, Zhao YL, et al. Home risk factors for childhood pneumonia in Nanjing, China. Chinese Sci Bull 2013;58:4230-6.

[14] Lu C, Deng Q H, Ou CY, et al. Effects of ambient air pollution on allergic rhinitis among preschool children in Changsha, China. Chinese Sci Bull.

2013;58:4252-8.
[15] Wang H, Li BZ, Yang Q, et al. Dampness in dwellings and its associations with asthma and allergies among children in Chongqing: A cross-sectional study. Chinese Sci Bull. 2013;58:4259-66.
[16] Wang J, Li BZ, Yang Q, et al. Sick building syndrome among parents of preschool children in relation to home environment in Chongqing, China. Chinese Sci Bull. 2013;58:4267-76.

Table S1: Age-specific and age-adjusted prevalence of pneumonia among preschool children in the Chinese seven cities in 2011 and 2019 by gender

	2011			2019		
	Boys	Girls	Total	Boys	Girls	Total
Age (years)						
2-4	34.3%(32.3-36.3)	29.1%(27.1-31.1)	31.7%(30.3-33.2)	29.1%(27.7-30.6)	25.6%(24.2-27.1)	27.5%(26.4-28.5)
4-6	35.7%(34.8-36.7)	32.8%(31.8-33.7)	34.3%(33.6-35.0)	29.1%(28.2-30.0)	26.7%(25.8-27.6)	27.9%(27.3-28.5)
6-8	33.8%(32.3-35.2)	30.1%(28.6-31.6)	32.0%(31.0-33.1)	29.7%(28.1-31.4)	26.1%(24.4-27.8)	28.0%(26.8-29.2)
p value for trend	0.3290	0.8560	0.4410	0.6318	0.5686	0.4490
Urbanisation						
Urban	35.6%(34.8-36.5)	31.7%(30.9-32.6)	33.7%(33.1-34.3)	29.0%(28.3-29.8)	26.0%(25.3-26.8)	27.6%(27.1-28.2)
Suburb	32.2%(30.6-33.8)	28.8%(27.2-30.4)	30.6%(29.4-31.7)	31.0%(29.2-32.8)	27.1%(25.3-28.9)	29.2%(27.9-30.5)
Rural	25.4%(21.9-29.1)	21.5%(18.0-25.4)	23.6%(21.1-26.2)	27.2%(23.7-30.8)	23.5%(19.8-27.5)	25.6%(23.0-28.2)
p value for difference	<0.0001	<0.0001	<0.0001	0.0680	0.2406	0.0169
Duration of breastfeeding						
＜6 months	37.8%(36.7-38.8)	32.9%(31.8-34.0)	35.4%(34.7-36.2)	31.3%(30.2-32.5)	27.4%(26.2-28.6)	29.5%(28.7-30.3)
≥6 months	31.5%(30.5-32.5)	28.5%(27.4-29.5)	30.0%(29.3-30.8)	28.0%(27.2-28.9)	25.4%(24.5-26.2)	26.7%(26.1-27.4)
p value for difference	<0.0001	<0.0001	<0.0001	<0.0001	0.0061	<0.0001
Premature birth						

Yes	35.4%(31.8-39.0)	34.5%(30.6-38.5)	32.6%(32.1-33.1)	29.1%(28.4-29.8)	34.7%(29.1-40.5)	36.2%(32.5-40.0)
No	34.5%(33.8-35.3)	30.5%(29.8-31.3)	34.9%(32.3-37.6)	37.5%(32.5-42.6)	26.0%(25.3-26.7)	27.6%(27.1-28.1)
p value for difference	0.6800	0.0468	0.0922	0.0006	0.0012	<0.0001
Birth weight (g)						
<2500	35.0%(31.0-39.0)	34.8%(31.4-38.4)	34.9%(32.3-37.6)	34.0%(30.1-38.1)	30.5%(26.8-34.5)	32.3%(29.6-35.1)
2500-4000	34.9%(34.1-35.7)	30.5%(29.8-31.3)	32.8%(32.2-33.3)	29.3%(28.5-30.1)	26.3%(25.5-27.1)	27.8%(27.3-28.4)
≥4000	31.1%(28.7-33.7)	29.0%(25.9-32.2)	30.3%(28.4-32.3)	28.6%(27.1-30.2)	24.1%(22.3-25.9)	26.9%(25.7-28.0)
p value for trend	0.0195	0.0143	0.0036	0.0566	0.0019	0.0030
Parental smoking						
None	33.0%(32.0-34.0)	30.2%(29.2-31.2)	31.6%(30.9-32.3)	27.1%(26.3-27.9)	24.7%(23.9-25.5)	25.9%(25.4-26.5)
One	36.4%(35.3-37.5)	31.2%(30.1-32.3)	33.9%(33.1-34.7)	34.7%(33.3-36.1)	29.6%(28.3-31.0)	32.3%(31.3-33.3)
Two	36.7%(27.0-47.3)	31.8%(21.9-43.1)	34.3%(27.3-41.8)	32.9%(21.8-45.6)	29.2%(17.4-43.4)	31.6%(23.4-40.8)
p value for trend	<0.0001	0.1650	<0.0001	<0.0001	<0.0001	<0.0001
Used antibiotics						
Yes	39.8%(39.0-40.7)	35.5%(34.7-36.4)	37.8%(37.2-38.4)	10.1%(9.2-11.0)	8.9%(8.0-9.8)	9.5%(8.9-10.2)
No	16.8%(15.6-18.0)	15.2%(14.1-16.4)	16.0%(15.2-16.9)	36.2%(35.4-37.1)	32.5%(31.7-33.4)	34.5%(33.9-35.1)
p value for difference	<0.0001	<0.0001	<0.0001	<0.0001	<0.0001	<0.0001
History of parental asthma						
None	34.2%(33.5-35.0)	30.3%(29.6-31.1)	32.3%(31.8-32.9)	28.9%(28.2-29.6)	25.9%(25.2-26.6)	27.5%(27.0-28.0)
One	45.6%(41.1-50.1)	41.9%(37.1-46.8)	43.9%(40.6-47.1)	47.3%(42.1-52.5)	35.8%(30.3-41.6)	42.2%(38.4-46.0)
Two	19.4%(1.2-62.9)	65.6%(37.3-87.5)	49.1%(27.5-71.0)	25.9%(13.1-42.6)	19.2%(9.2-33.4)	22.3%(13.9-32.6)
p value for trend	<0.0001	<0.0001	<0.0001	<0.0001	0.0204	<0.0001
History of parental allergy						
None	33.1%(32.4-33.9)	29.5%(28.7-30.3)	31.4%(30.8-31.9)	26.2%(25.4-27.0)	23.2%(22.4-24.0)	24.8%(24.2-25.3)
One	46.3%(43.8-48.8)	41.0%(38.4-43.6)	43.8%(42.0-45.6)	36.1%(34.6-37.5)	32.7%(31.2-34.3)	34.5%(33.5-35.6)
Two	55.4%(47.4-63.2)	41.2%(33.0-49.8)	48.6%(42.9-54.4)	39.0%(35.5-42.5)	35.5%(32.0-39.1)	37.3%(34.8-39.8)

p value for trend	<0.0001	<0.0001	<0.0001	<0.0001	<0.0001	<0.0001
Residence area						
＜75m^2	34.3%(33.2-35.5)	29.6%(28.5-30.8)	32.1%(31.2-32.9)	29.2%(27.8-30.6)	25.3%(23.9-26.8)	27.3%(26.3-28.3)
⩾75m^2	34.8%(33.8-35.7)	31.3%(30.3-32.2)	33.1%(32.4-33.8)	29.4%(28.6-30.2)	26.4%(25.6-27.2)	27.9%(27.4-28.5)
p value for difference	0.5830	0.0348	0.0609	0.8401	0.2296	0.2811
Frequency of putting bedding to sunshine						
Never	38.3%(34.0-42.8)	34.0%(29.9-38.3)	36.2%(33.2-39.2)	26.5%(22.4-30.8)	23.8%(19.7-28.3)	25.1%(22.2-28.2)
Sometimes	37.0%(35.7-38.4)	34.1%(32.8-35.5)	35.6%(34.7-36.6)	32.6%(31.4-33.8)	28.7%(27.5-29.9)	30.7%(29.9-31.6)
Often	33.2%(32.3-34.1)	28.7%(27.8-29.6)	31.0%(30.4-31.6)	27.6%(26.8-28.5)	24.8%(24.0-25.7)	26.3%(25.7-26.9)
p value for trend	<0.0001	<0.0001	<0.0001	<0.0001	0.0001	<0.0001
Frequency of clean children's rooms						
Rarely	32.2%(26.1-38.8)	29.3%(23.3-36.0)	30.8%(26.5-35.4)	30.8%(26.5-35.4)	28.6%(24.1-33.4)	29.8%(26.7-33.0)
Sometimes	35.9%(34.0-37.8)	32.9%(31.0-34.8)	34.4%(33.1-35.8)	31.8%(30.2-33.5)	28.3%(26.6-29.9)	30.1%(29.0-31.3)
Often	34.4%(33.6-35.2)	30.3%(29.5-31.1)	32.4%(31.8-33.0)	28.7%(27.9-29.5)	25.5%(24.8-26.3)	27.2%(26.6-27.7)
p value for trend	0.4010	0.0550	0.0533	0.0015	0.0022	<0.0001
Cooking fuel type						
Natural gas/coal gas	35.3%(34.5-36.2)	31.3%(30.5-32.2)	33.4%(32.8-34.0)	29.7%(29.0-30.4)	26.3%(25.6-27.0)	28.1%(27.6-28.6)
Coal	37.2%(34.1-40.3)	30.5%(27.6-33.6)	33.9%(31.8-36.1)	34.7%(23.1-47.9)	37.6%(23.7-53.3)	36.9%(27.8-46.8)
Electricity	28.9%(26.9-30.9)	26.2%(24.3-28.3)	27.6%(26.2-29.0)	23.9%(20.8-27.1)	24.1%(21.0-27.4)	24.0%(21.8-26.2)
Wood	27.8%(18.8-38.3)	24.0%(15.8-33.9)	25.8%(19.7-32.8)	23.3%(3.7-59.0)	33.3%(1.9-85.5)	29.2%(8.7-58.7)
Other	36.5%(33.0-40.1)	33.3%(29.7-37.0)	34.9%(32.4-37.5)	10.0%(5.2-17.0)	12.2%(6.1-21.2)	11.0%(7.0-16.2)
p value for difference	<0.0001	0.0001	<0.0001	<0.0001	0.0099	<0.0001
Indoor dampness						
Yes	41.4%(39.3-43.5)	37.5%(35.3-39.6)	39.5%(38.0-41.0)	35.3%(33.4-37.3)	32.2%(30.3-34.2)	33.9%(32.5-35.2)

No	33.6%(32.8-34.3)	29.7%(28.9-30.4)	31.7%(31.1-32.2)	28.3%(27.6-29.1)	25.1%(24.4-25.9)	26.8%(26.3-27.3)
p value for difference	<0.0001	<0.0001	<0.0001	<0.0001	<0.0001	<0.0001
Home interior decoration						
Yes	37.7%(35.9-39.5)	34.0%(32.1-35.8)	35.9%(34.6-37.2)	27.7%(25.7-29.8)	24.4%(22.3-26.6)	26.2%(24.7-27.7)
No	33.9%(33.1-34.7)	29.9%(29.1-30.7)	32.0%(31.4-32.6)	29.5%(28.7-30.2)	26.3%(25.6-27.1)	28.0%(27.4-28.5)
p value for difference	0.0001	<0.0001	0.0001	0.1217	0.0914	0.0265
Home interior purchase new furniture						
Yes	36.2%(34.9-37.4)	33.0%(31.7-34.3)	31.7%(31.0-32.3)	29.3%(27.9-30.8)	24.9%(23.5-26.5)	27.3%(26.2-28.4)
No	33.8%(32.9-34.7)	29.4%(28.5-30.3)	34.6%(33.7-35.5)	29.2%(28.5-30.0)	26.4%(25.6-27.2)	27.9%(27.3-28.4)
p value for difference	0.0024	<0.0001	<0.0001	0.9544	0.0898	0.3308
Wall painting materials						
Wall paper	31.4%(29.1-33.8)	33.2%(30.8-35.7)	32.3%(30.6-34.0)	28.2%(26.8-29.6)	26.3%(24.9-27.7)	27.3%(26.3-28.3)
Emulsion paint	36.4%(35.4-37.3)	31.2%(30.2-32.1)	33.8%(33.1-34.5)	31.1%(30.0-32.3)	26.8%(25.7-28.0)	29.1%(28.3-29.9)
Paint	38.0%(35.7-40.3)	33.0%(30.7-35.3)	35.6%(34.0-37.2)	27.0%(22.9-31.5)	28.6%(23.8-33.8)	27.7%(24.6-31.0)
Wood	22.2%(16.0-29.5)	23.9%(17.7-30.9)	23.0%(18.6-27.9)	12.2%(7.0-19.4)	17.1%(9.7-27.0)	14.5%(10.0-20.1)
Lime / cement	29.8%(28.0-31.7)	27.1%(25.2-29.0)	28.6%(27.3-29.9)	24.0%(19.7-28.6)	24.8%(20.3-29.8)	24.4%(21.3-27.7)
Other	30.4%(27.3-33.6)	27.1%(24.2-30.3)	28.9%(26.7-31.1)	28.7%(27.4-29.9)	25.1%(23.8-26.4)	27.0%(26.1-27.9)
p value for difference	<0.0001	<0.0001	<0.0001	<0.0001	0.1028	<0.0001
Flooring materials						
Solid wood / multi-layer solid wood	34.0%(32.9-35.1)	30.2%(29.1-31.2)	32.1%(31.4-32.9)	27.8%(26.7-28.9)	26.5%(25.4-27.6)	27.2%(26.4-27.9)
Laminate / Composite wood	38.9%(37.3-40.5)	34.8%(33.2-36.5)	37.0%(35.9-38.1)	31.8%(30.6-33.1)	27.6%(26.3-28.8)	29.8%(28.9-30.7)
Bamboo	32.4%(26.3-38.9)	27.3%(21.5-33.6)	30.0%(25.8-34.5)	27.1%(19.8-35.5)	20.5%(14.2-28.0)	23.9%(19.0-29.3)
Ceramic tile / stone / cement	32.2%(30.8-33.6)	28.2%(26.8-29.7)	30.4%(29.4-31.4)	29.2%(27.8-30.6)	24.4%(23.1-25.7)	26.9%(25.9-27.8)
PVC / plastics / plastic	37.9%(31.2-45.1)	21.8%(16.1-28.3)	28.9%(24.5-33.7)	29.4%(19.1-41.4)	24.6%(11.2-43.0)	27.9%(19.4-37.6)

leather						
Other	32.3%(26.7-38.4)	29.7%(24.2-35.6)	30.9%(26.9-35.0)	20.8%(16.4-25.7)	22.8%(17.8-28.3)	21.7%(18.3-25.3)
p value for difference	<0.0001	<0.0001	<0.0001	<0.0001	0.0074	<0.0001
Indoor heating mode						
No heating	34.0%(31.9-36.0)	27.6%(25.7-29.7)	31.0%(29.5-32.4)	28.6%(27.4-29.9)	26.1%(24.8-27.5)	27.5%(26.6-28.4)
Individual household heating	34.0%(33.1-34.9)	30.8%(29.9-31.7)	32.4%(31.8-33.1)	30.5%(29.5-31.6)	26.6%(25.6-27.6)	28.7%(27.9-29.4)
Central heating	38.0%(36.4-39.7)	33.0%(31.3-34.7)	35.7%(34.5-36.9)	28.1%(26.6-29.6)	25.2%(23.8-26.7)	26.7%(25.6-27.7)
Other	29.1%(23.7-35.0)	29.0%(23.8-34.7)	29.1%(25.3-33.1)	26.5%(23.1-30.1)	24.9%(21.3-28.7)	25.7%(23.2-28.3)
p value for difference	<0.0001	0.0010	<0.0001	0.0098	0.4250	0.0052
Indoor use air-conditioning						
Yes	33.6%(32.8-34.5)	30.7%(29.8-31.6)	33.4%(32.5-34.3)	29.6%(28.8-30.4)	26.4%(25.6-27.2)	28.1%(27.5-28.6)
No	36.2%(35.0-37.6)	30.1%(28.8-31.4)	32.2%(31.6-32.8)	28.0%(26.5-29.6)	25.0%(23.5-26.6)	26.6%(25.5-27.7)
p value for difference	0.0011	0.5000	0.0327	0.0789	0.1334	0.0193
Indoor use air purifier						
Yes	36.3%(33.0-39.6)	32.9%(29.7-36.3)	34.7%(32.4-37.1)	29.5%(28.4-30.7)	26.9%(25.8-28.1)	28.3%(27.5-29.1)
No	34.5%(33.7-35.2)	30.5%(29.8-31.3)	32.6%(32.0-33.1)	29.2%(28.3-30.1)	25.8%(24.9-26.6)	27.6%(26.9-28.2)
p value for difference	0.3040	0.1600	0.0780	0.6483	0.1210	0.1681
Indoor keeping furry pets						
Yes	34.4%(32.2-36.6)	29.7%(27.6-31.9)	32.1%(30.6-33.7)	30.2%(28.1-32.4)	28.7%(26.7-30.8)	29.4%(28.0-30.9)
No	34.6%(33.9-35.4)	30.8%(30.0-31.6)	32.8%(32.3-33.4)	29.2%(28.5-29.9)	25.7%(25.0-26.5)	27.6%(27.1-28.1)
p value for difference	0.8370	0.3520	0.4220	0.4000	0.0062	0.0179
Asthma						
Yes	62.2%(59.7-64.6)	62.3%(59.1-65.4)	62.2%(60.3-64.1)	59.1%(55.5-62.6)	60.0%(55.3-64.5)	59.5%(56.6-62.2)
No	31.9%(31.1-32.6)	28.7%(27.9-29.4)	30.3%(29.8-30.8)	27.9%(27.2-28.6)	25.0%(24.3-25.7)	26.5%(26.0-27.0)
p value for difference	<0.0001	<0.0001	<0.0001	<0.0001	<0.0001	<0.0001
Allergic rhinitis						

Yes	49.6%(47.3-51.9)	44.8%(42.0-47.5)	47.6%(45.9-49.4)	44.5%(42.4-46.6)	43.5%(41.0-46.0)	44.1%(42.5-45.7)
No	32.7%(32.0-33.5)	29.4%(28.6-30.2)	31.1%(30.6-31.6)	27.0%(26.3-27.8)	24.2%(23.5-24.9)	25.6%(25.1-26.2)
p value for difference	<0.0001	<0.0001	<0.0001	<0.0001	<0.0001	<0.0001
Wheezing						
Yes	48.2%(46.8-49.7)	45.7%(44.1-47.4)	47.1%(46.0-48.2)	57.6%(55.0-60.3)	57.1%(53.7-60.5)	57.3%(55.2-59.4)
No	29.9%(29.0-30.7)	26.9%(26.1-27.7)	28.4%(27.8-29.0)	26.8%(26.0-27.5)	24.3%(23.6-25.0)	25.6%(25.1-26.1)
p value for difference	<0.0001	<0.0001	<0.0001	<0.0001	<0.0001	<0.0001

Values are % (95% CI). p value for difference refers to the comparison of binary variables. Pneumonia, asthma, allergic rhinitis were defined as physician-diagnosed since birth of children. Wheezing was defined as parents reported that the child has great difficulty breathing or the respiratory muscles of the child are all involved in breathing, and the respiratory rate of the child is faster than normal since birth. Premature birth was defined as delivery under 37 weeks of pregnancy. Used antibiotics was defined as child has been injected or taken since birth, such as but not limited to penicillin, azithromycin, cephalosporin, etc. Parental smoking was defined as parents smoked equal to or more than 100 cigarettes in the lifetime. All calculations of p values are weighted, accounting for the number of people sampled in two surveys in 2011 and 2019 and based on the χ^2 test.

Table S2: Multiple adjusted odds ratios of pneumonia in the Chinese seven cities of preschool children in 2011 and 2019 by duration of breastfeeding and antibiotics use

	Duration of breastfeeding ≥ 6 months		Never used antibiotics		Used antibiotics		Duration of breastfeeding ≥ 6 months		Never used antibiotics		Used antibiotics	
	OR(95%CI)	p value	OR(95%CI)	p value	OR(95%CI)	p value	OR(95%CI)	p value	OR(95%CI)	p value	OR(95%CI)	p value
Girls	0.93(0.86-0.99)	0.0350	0.94(0.83-1.07)	0.3437	0.91(0.86-0.96)	0.0004	0.96(0.90-1.02)	0.1969	0.92(0.87-0.97)	0.0025	0.93(0.80-1.09)	0.3745
Age							..	..	..	..	..	..
2-4	1.00		1.00		1.00		..	..	..	..	..	..
4-6	1.13(1.02-1.	0.02	1.15(0.94-1.	0.17	1.11(1.02-1.	0.01	..	..	..	..	..	..

	26)	56	42)	16	20)	31						
6-8	0.95(0.84-1.07)	0.4057	0.92(0.73-1.16)	0.4909	0.99(0.90-1.09)	0.8181	..	..	..	..	..	..
Urbanisation												
Urban	1.00		1.00		1.00		1.00		1.00		1.00	
Suburb	0.97(0.89-1.06)	0.5327	1.02(0.86-1.19)	0.860	0.94(0.88-1.01)	0.1100	1.10(1.00-1.20)	0.0465	1.09(1.01-1.18)	0.0229	1.15(0.95-1.40)	0.1500
Rural	1.00(0.81-1.22)	0.9841	1.09(0.80-1.45)	0.5813	0.78(0.65-0.93)	0.0073	1.15(0.95-1.38)	0.1348	1.15(0.97-1.36)	0.0956	1.09(0.77-1.51)	0.5979
Duration of breastfeeding ≥ 6 months	..	..	0.85(0.75-0.97)	0.0164	0.83(0.79-0.88)	＜0.0001	..	..	0.92(0.86-0.97)	0.0028	0.96(0.82-1.12)	0.5886
Premature birth	..	..	..	..	..	..	1.32(1.02-1.70)	0.0323	1.28(1.05-1.55)	0.0128	1.34(0.77-2.21)	0.2693
Birth weight (g)												
＜2500	0.99(0.81-1.21)	0.9351	0.85(0.60-1.17)	0.3212	1.16(1.02-1.32)	0.0285	1.21(0.99-1.47)	0.0551	1.24(1.06-1.44)	0.0072	0.83(0.54-1.24)	0.3890
2500~4000	1.00		1.00		1.00		1.00		1.00		1.00	
≥4000	0.93(0.81-1.06)	0.2817	0.90(0.70-1.16)	0.4309	0.88(0.79-0.98)	0.0186	0.87-1.04	0.2466	0.98(0.90-1.05)	0.5407	0.92(0.76-1.12)	0.4322
Parental smoking												
None	1.00		1.00		1.00		1.00		1.00		1.00	
One	1.13(1.05-1.21)	0.0010	1.31(1.15-1.49)	＜0.0001	1.09(1.03-1.15)	0.0015	1.34(0.25-1.44)	＜0.0001	1.27(1.19-1.35)	＜0.0001	1.53(1.30-1.79)	＜0.0001
Two	0.91(0.52-1.	0.73	0.99(0.42-2.	0.97	1.04(0.71-1.	0.84	1.28(0.74-2.	0.362	1.30(0.82-2.	0.24	1.92(0.71-4.	0.160

	54)	43	03)	23	51)	19	13)	5	04)	99	50)	5
Used antibiotics	2.66(2.41-2.93)	<0.0001	..	..	..	..	0.23(0.20-0.25)	<0.0001	..	..	..	..
History of parental asthma	..	..	..	..	..	..						
None	..	..	..	..	..	..	1.00		1.00		1.00	
One	..	..	..	..	..	..	1.24(0.98-1.55)	0.0678	1.20(1.00-1.45)	0.0463	1.38(0.81-2.24)	0.2148
Two	..	..	..	..	..	..	1.20(0.66-2.13)	0.5423	1.00(0.60-1.61)	0.9880	0.76(0.04-4.26)	0.7551
History of parental allergy												
None	1.00		1.00		1.00		1.00		1.00		1.00	
One	1.15(1.02-1.31)	0.0261	1.37(1.05-1.79)	0.0195	1.19(1.09-1.30)	<0.0001	1.20(1.11-1.30)	<0.0001	1.20(1.13-1.28)	<0.0001	1.12(0.92-1.35)	0.2551
Two	1.84(1.28-2.66)	0.0010	2.37(1.22-4.53)	0.0096	1.20(0.93-1.55)	0.1614	1.17(1.00-1.36)	0.0454	1.17(1.03-1.33)	0.0140	1.83(1.26-2.61)	0.0011
Frequency of putting bedding to sunshine							..	..	..	..	..	..
Never	1.00		1.00		1.00		..	..	..	..	..	..
Sometimes	0.98(0.80-1.21)	0.8757	0.82(0.59-1.14)	0.2275	0.94(0.80-1.10)	0.4497	..	..	..	..	..	..
Often	0.87(0.71-1.08)	0.2003	0.67(0.49-0.93)	0.0142	0.85(0.73-0.99)	0.0424	..	..	..	..	..	..

Cooking fuel type												
Natural gas/coal gas	1.00		1.00		1.00		1.00		1.00		1.00	
Coal	0.93(0.79-1.10)	0.4231	0.86(0.66-1.12)	0.2851	1.03(0.92-1.16)	0.5774	1.83(1.01-3.21)	0.0398	1.33(0.80-2.18)	0.2546	2.52(1.07-5.35)	0.0233
Electricity	0.83(0.74-0.93)	0.0019	0.92(0.76-1.11)	0.3750	0.86(0.78-0.93)	0.0005	1.03(0.88-1.22)	0.6864	0.98(0.85-1.13)	0.7667	0.83(0.58-1.15)	0.8295
Wood	0.67(0.39-1.09)	0.1182	0.80(0.36-1.59)	0.5463	0.82(0.55-1.21)	0.3252	--	0.9035*	0.69(0.12-3.27)	0.6547	--	0.9577*
Other	1.05(0.89-1.24)	0.5473	0.97(0.70-1.31)	0.8422	1.15(1.01-1.31)	0.0331	0.33(0.14-0.66)	0.0036	0.45(0.25-0.76)	0.0050	0.28(0.07-0.75)	0.0311
Indoor dampness	1.24(1.12-1.38)	＜0.0001	1.34(1.10-1.63)	0.0028	1.22(1.13-1.32)	＜0.0001	1.20(1.09-1.31)	0.0001	1.16(1.07-1.25)	0.0002	1.13(0.90-1.41)	0.2880
Home interior decoration	1.02(0.93-1.12)	0.6747	1.15(0.96-1.38)	0.1234	1.11(1.03-1.19)	0.0042	..	..	..	..	..	..
Wall painting materials							..	..	..	..	..	..
Wall paper			1.00		1.00		..	..	..	..	..	..
Emulsion paint	1.09(0.96-1.25)	0.1764	1.09(0.88-1.37)	0.4229	1.06(0.96-1.16)	0.2399	..	..	..	..	..	..
Paint	1.16(0.98-1.25)	0.0910	0.99(0.74-1.31)	0.9283	1.19(1.06-1.34)	0.0043	..	..	..	..	..	..
Wood	0.92(0.62-1.34)	0.6784	0.54(0.28-0.97)	0.0529	0.95(0.69-1.29)	0.7296	..	..	..	..	..	..
Lime / cement	0.98(0.84-1.15)	0.8031	1.04(0.80-1.35)	0.7621	0.98(0.87-1.10)	0.6849	..	..	..	..	..	..
Other	0.99(0.81-1.	0.93	0.85(0.61-1.	0.33	0.97(0.83-1.	0.65	..	..	..	..	..	..

	21)	20	18)	51	13)	70						
Flooring materials							..	..	..	..	..	..
Solid wood / multi-layer solid wood	1.00		1.00		1.00		..	..	..	..	..	..
Laminate / Composite wood	1.07(0.97-1.18)	0.1642	1.15(0.97-1.38)	0.1123	1.07(1.00-1.15)	0.0459	..	..	..	..	..	..
Bamboo	0.86(0.63-1.15)	0.3215	0.96(0.53-1.63)	0.8886	0.79(0.62-0.99)	0.0444	..	..	..	..	..	..
Ceramic tile / stone / cement	0.96(0.87-1.05)	0.3845	0.96(0.81-1.14)	0.6592	1.01(0.94-1.09)	0.7251	..	..	..	..	..	..
PVC / plastics / plastic leather	0.83(0.59-1.14)	0.2578	1.31(0.76-2.16)	0.3020	0.90(0.70-1.16)	0.4270	..	..	..	..	..	..
Other	1.17(0.88-1.54)	0.2653	0.90(0.57-1.37)	0.6272	1.15(0.91-1.44)	0.2338	..	..	..	..	..	..
Indoor heating mode							..	..	..	..	..	..
No heating	1.00		1.00		1.00		..	..	..	..	..	..
Individual household heating	1.05(0.93-1.17)	0.4391	1.02(0.84-1.25)	0.8218	1.04(0.96-1.14)	0.3300	..	..	..	..	..	..
Central heating	1.18(1.04-1.35)	0.0134	1.06(0.84-1.35)	0.6179	1.20(1.08-1.33)	0.0007	..	..	..	..	..	..
Other	0.83(0.61-1.11)	0.2184	0.92(0.57-1.43)	0.7179	1.08(0.86-1.36)	0.5120	..	..	..	..	..	..
Indoor use air-conditioning	0.82(0.76-0.90)	＜0.0001	0.84(0.72-0.98)	0.0215	0.85(0.79-0.91)	＜0.0001	0.87(0.80-0.95)	0.0017	0.88(0.82-0.95)	0.0008	0.95(0.80-1.13)	0.5484
Asthma	2.66(2.30-3.	＜	3.89(2.98-5.	＜	2.24(2.03-2.	＜	2.05(1.71-2.	＜	1.72(1.50-1.	＜	4.47(2.90-6.	＜

	07)	0.0001	09)	0.0001	47)	0.0001	46)	0.0001	98)	0.0001	84)	0.0001
Allergic rhinitis	1.41(1.24-1.60)	＜0.0001	1.30(1.00-1.68)	0.0482	1.37(1.25-1.49)	＜0.0001	1.54(0.39-1.71)	＜0.0001	1.56(1.44-1.69)	＜0.0001	1.56(1.17-2.05)	0.0018
Wheezing	1.69(1.55-1.83)	＜0.0001	1.90(1.62-2.22)	＜0.0001	1.61(1.51-1.71)	＜0.0001	2.40(2.10-2.75)	＜0.0001	2.41(2.17-2.67)	＜0.0001	2.75(1.91-3.89)	＜0.0001

After a multivariate logistic stepwise regression analysis, the co-variates that were finally included in the analysis are listed in the table. ORs of 1.00 indicate reference values. Pneumonia, asthma, allergic rhinitis were defined as physician-diagnosed since birth of children. Wheezing was defined as parents reported that the child has great difficulty breathing or the respiratory muscles of the child are all involved in breathing, and the respiratory rate of the child is faster than normal since birth. Premature birth was defined as delivery under 37 weeks of pregnancy. Used antibiotics was defined as child has been injected or taken since birth, such as but not limited to penicillin, azithromycin, cephalosporin, etc. Parental smoking was defined as parents smoked equal to or more than 100 cigarettes in the lifetime. OR=odds ratio. [*]p value were OR ＜0.0001 maybe due to too few subjects for these co-variates.

Table S3: Multiple adjusted odds ratios of asthma, allergic rhinitis and wheezing and in the Chinese seven cities of preschool children in 2011 and 2019

	2011						2019					
	Asthma		Allergic rhinitis		Wheezing		Asthma		Allergic rhinitis		Wheezing	
	OR(95%CI)	p value	OR(95%CI)	p value	OR(95%CI)	p value	OR(95%CI)	p value	OR(95%CI)	p value	OR(95%CI)	p value
Girls	0.74(0.67-0.81)	<0.0001	0.75(0.69-0.81)	<0.0001	0.86(0.81-0.91)	<0.0001	0.81(0.71-0.93)	0.0022	0.81(0.75-0.87)	<0.0001	0.72(0.65-0.80)	<0.0001
Age												
2-4	..	..	1.00		1.00		..	..	..	..	..	..
4-6	..	..	1.60(1.39-	<0.0	1.14(1.04-	0.00	..	..	..	..	..	..

			1.86)	001	1.25)	47						
6-8	..	..	2.25(1.93-2.64)	<0.0001	2.24(2.04-2.47)	<0.0001	..	..	..	..	..	..
Urbanisation												
Urban	1.00		1.00		1.00		1.00		1.00		..	..
Suburb	0.85(0.75-0.95)	0.0056	0.84(0.76-0.93)	0.0013	1.14(1.07-1.22)	0.0001	1.34(1.13-1.58)	0.0007	0.82(0.73-0.91)	0.0002	..	..
Rural	0.63(0.44-0.88)	0.0096	0.44(0.29-0.62)	<0.0001	0.90(0.76-1.05)	0.1910	1.16(0.78-1.67)	0.4443	0.69(0.52-0.90)	0.0072	..	..
Duration of breastfeeding ≥6 months	0.84(0.76-0.92)	0.0002	0.85(0.78-0.92)	<0.0001	1.05(1.00-1.11)	0.0724	0.87(0.77-1.00)	0.0437	0.80(0.75-0.87)	<0.0001	0.81(0.73-0.89)	<0.0001
Birth weight (g)												
＜2500	..	..	..	..	..	..	..	..	1.00		..	..
2500~4000	..	..	..	..	..	..	..	..	1.30(1.06-1.62)	0.0152	..	..
≥4000	..	..	..	..	..	..	..	..	1.35(1.08-1.70)	0.011	..	..
Parental smoking												
None	..	..	..	..	..	..	..	..	1.00		1.00	
One	..	..	..	..	..	..	..	..	1.10(1.01-1.19)	0.0284	1.16(1.04-1.29)	0.0072
Two	..	..	..	..	..	..	..	..	0.53(0.47-0.58)	<0.0001	1.85(0.95-3.36)	0.0570
Used antibiotics	1.46(1.27-1.69)	<0.0001	1.61(1.44-1.82)	<0.0001	1.46(1.36-1.57)	<0.0001	0.62(0.50-0.77)	<0.0001	0.53(0.47-0.58)	<0.0001	0.47(0.40-0.55)	<0.0001

History of parental asthma												
None	1.00		1.00		1.00		1.00		1.00		1.00	
One	2.67(2.23-3.18)	<0.0001	1.34(1.11-1.59)	0.0016	2.00(1.72-2.32)	<0.0001	2.95(2.29-3.80)	<0.0001	0.96(0.78-1.18)	0.7060	2.71(2.16-3.38)	<0.0001
Two	5.81(2.11-15.58)	0.0005	1.53(0.47-4.19)	0.4391	3.23(1.21-9.20)	0.022	1.48(0.42-3.92)	0.4812	0.20(0.08-0.41)	<0.0001	0.44(0.12-1.16)	0.1427
History of parental allergy												
None	1.00		1.00		1.00		1.00		1.00		1.00	
One	1.32(1.17-1.50)	<0.0001	4.11(3.73-4.52)	<0.0001	1.41(1.29-1.55)	<0.0001	1.23(1.07-1.42)	0.0040	3.00(2.78-3.24)	<0.0001	1.48(1.33-1.65)	<0.0001
Two	1.22(0.87-1.69)	0.2503	7.04(5.49-9.00)	<0.0001	1.85(1.44-2.39)	<0.0001	1.30(1.00-1.67)	0.044	5.56(4.89-6.32)	<0.0001	1.62(1.33-1.96)	<0.0001
Residence area ⩾ 75m^2	..	..	1.16(1.06-1.26)	0.0010	1.10(1.04-1.16)	0.0013	..	..				
Frequency of clean children's rooms												
Rarely	..	..	..	..	1.00		..	..	..	..	1.00	
Sometimes	..	..	..	..	0.92(0.73-1.17)	0.5054	..	..	..	..	0.78(0.60-1.03)	0.0725
Often	..	..	..	..	0.79(0.63-0.99)	0.0401	..	..	..	..	0.67(0.52-0.87)	0.0020
Indoor dampness	..	..	1.20(1.07-1.33)	0.0011	1.32(1.22-1.43)	<0.0001	..	..	1.12(1.02-1.24)	0.0159	1.42(1.26-1.60)	<0.0001
Home interior	..	..	..	..	1.11(1.05-	0.00	..	..	..	..	..	..

purchase new furniture					1.18)	03						
Wall painting materials												
Wall paper	..	..	1.00		..	..	..	..	1.00		..	..
Emulsion paint	..	..	1.05(0.92-1.20)	0.5004	..	..	..	..	1.01(0.93-1.11)	0.7692	..	..
Paint	..	..	1.09(0.92-1.29)	0.3078	..	..	..	..	0.74(0.56-0.96)	0.0285	..	..
Wood	..	..	0.84(0.52-1.30)	0.4528	..	..	..	..	0.96(0.55-1.59)	0.8925	..	..
Lime / cement	..	..	0.81(0.67-0.97)	0.0242	..	..	..	..	0.98(0.72-1.29)	0.8641	..	..
Other	..	..	0.82(0.64-1.04)	0.1048	..	..	..	..	0.83(0.75-0.92)	0.0006	..	..
Flooring materials												
Solid wood / multi-layer solid wood	1.00		1.00		..	..	1.00		1.00		1.00	
Laminate / Composite wood	0.91(0.81-1.01)	0.0852	1.04(0.95-1.15)	0.4142	..	..	0.87(0.75-1.01)	0.0706	1.14(1.05-1.24)	0.0017	1.06(0.95-1.19)	0.2862
Bamboo	1.09(0.73-1.60)	0.6626	1.12(0.80-1.54)	0.4885	..	..	1.08(0.50-2.11)	0.8249	1.05(0.67-1.57)	0.8342	1.10(0.62-1.84)	0.7280
Ceramic tile / stone / cement	0.77(0.67-0.88)	0.0001	0.74(0.65-0.84)	<0.0001	..	..	0.96(0.79-1.15)	0.6491	0.79(0.71-0.88)	<0.0001	0.75(0.65-0.87)	<0.0001
PVC / plastics /	0.83(0.53-	0.41	0.61(0.38-	0.02	..	..	0.18(0.01-0.	0.10	1.36(0.70-	0.33	1.30(0.52-2.	0.53

plastic leather	1.27)	79	0.92)	55			94)	49	2.48)	41	74)	18
Other	0.98(0.67-1.40)	0.9019	0.72(0.49-1.03)	0.0801	..	..	0.88(0.50-1.47)	0.6530	0.80(0.56-1.10)	0.1802	1.03(0.68-1.52)	0.8747
Indoor heating mode												
No heating	1.00		..	..	..	..	1.00		1.00		1.00	
Individual household heating	0.88(0.77-1.01)	0.0755	..	..	..	..	0.88(0.76-1.02)	0.0849	1.05(0.96-1.14)	0.2858	0.97(0.87-1.09)	0.6117
Central heating	0.48(0.39-0.57)	<0.0001	..	..	..	..	0.53(0.42-0.67)	<0.0001	0.65(0.57-0.74)	<0.0001	0.74(0.62-0.87)	0.0005
Other	1.19(0.83-1.69)	0.3373	..	..	..	..	0.95(0.63-1.40)	0.8102	0.99(0.79-1.23)	0.9290	0.50(0.35-0.71)	0.0002
Indoor use air-conditioning	1.32(1.17-1.49)	<0.0001	..	..	..	..	..	..	1.26(1.11-1.42)	0.0003	0.83(0.71-0.98)	0.0219
Indoor use air purifier	..	..	1.42(1.22-1.65)	<0.0001	1.29(1.15-1.46)	<0.0001	..	..	1.26(1.16-1.35)	<0.0001	..	..
Indoor keeping furry pets	..	..	..	..	1.13(1.04-1.23)	0.0040	..	..	..	..	..	..
Asthma	..	..	2.78(2.49-3.11)	<0.0001	7.48(6.78-8.26)	<0.0001	..	..	1.84(1.58-2.14)	<0.0001	17.97(15.70-20.58)	<0.0001
Allergic rhinitis	2.75(2.46-3.07)	<0.0001	..	..	1.45(1.33-1.58)	<0.0001	1.84(1.58-2.14)	<0.0001	..	..	1.81(1.60-2.04)	<0.0001
Wheezing	7.34(6.66-8.10)	<0.0001	1.48(1.36-1.62)	<0.0001	..	..	17.98(15.71-20.60)	<0.0001	1.80(1.59-2.02)	<0.0001	..	..
Pneumonia	2.53(2.31-2.78)	<0.0001	1.41(1.29-1.53)	<0.0001	1.64(1.55-1.74)	<0.0001	1.96(1.72-2.25)	<0.0001	1.60(1.48-1.73)	<0.0001	2.49(2.25-2.75)	<0.0001

After a multivariate logistic stepwise regression analysis, the co-variates that were finally included in the analysis are listed in the table. ORs of 1.00 indicate reference values. Pneumonia, asthma, allergic rhinitis were defined as physician-diagnosed since birth of children. Wheezing was defined as parents reported that the child has great difficulty breathing or the respiratory muscles of the child are all involved in breathing, and the respiratory rate of the child is faster than normal since birth. Premature birth was defined as delivery under 37 weeks of pregnancy. Used antibiotics was defined as child has been injected or taken since birth, such as but not limited to penicillin, azithromycin, cephalosporin, etc. Parental smoking was defined as parents smoked equal to or more than 100 cigarettes in the lifetime. OR=odds ratio. *p value were OR ＜0.0001 maybe due to too few subjects for these co-variates.

Table S4: Age-specific and age-adjusted prevalence of pneumonia among preschool children in the Chinese seven cities in 2011 and 2019 by duration of breastfeeding

	2011			2019		
	Duration of breastfeeding ＜ 6 months	Duration of breastfeeding ≥ 6 months	Total	Duration of breastfeeding ＜ 6 months	Duration of breastfeeding ≥ 6 months	Total
Age (years)						
2-4	34.3%(32.2-36.4)	29.5%(27.6-31.4)	31.7%(30.3-33.2)	28.9%(27.2-30.6)	26.6%(25.3-27.9)	27.5%(26.4-28.5)
4-6	36.5%(35.6-37.5)	31.9%(30.9-32.8)	34.3%(33.6-35.0)	30.1%(29.1-31.2)	26.6%(25.8-27.4)	27.9%(27.3-28.5)
6-8	35.4%(33.9-37.0)	28.8%(27.4-30.2)	32.0%(31.0-33.1)	29.4%(27.5-31.4)	27.1%(25.6-28.6)	28.0%(26.8-29.2)
p value for difference	0.7315	0.1533	0.4410	0.6289	0.6632	0.4490
Gender						
Boys	37.8%(36.7-38.8)	31.5%(30.5-32.5)	34.6%(33.8-35.3)	31.3%(30.2-32.5)	28.0%(27.2-28.9)	29.3%(28.6-30.0)
Girls	32.9%(31.8-34.0)	28.5%(27.4-29.5)	30.6%(29.9-31.4)	27.4%(26.2-28.6)	25.4%(24.5-26.2)	26.1%(25.4-26.8)
p value for difference	<0.0001	<0.0001	<0.0001	<0.0001	<0.0001	<0.0001
Urbanisation						
Urban	36.4%(35.5-37.2)	31.0%(30.1-31.8)	33.7%(33.1-34.3)	29.3%(28.4-30.2)	26.5%(25.9-27.2)	27.6%(27.1-28.2)
Suburb	32.4%(30.8-34.1)	28.9%(27.4-30.4)	30.6%(29.4-31.7)	31.4%(29.2-33.6)	28.1%(26.5-29.6)	29.2%(27.9-30.5)

Rural	26.3%(22.2-30.8)	21.8%(18.7-25.1)	23.6%(21.1-26.2)	26.0%(21.8-30.5)	25.3%(22.2-28.7)	25.6%(23.0-28.2)
p value for difference	<0.0001	<0.0001	<0.0001	0.0601	0.1387	0.0169
Premature birth						
Yes	37.7%(34.4-41.1)	29.7%(25.5-34.0)	32.6%(32.1-33.1)	38.7%(33.4-44.3)	33.8%(28.7-39.2)	36.2%(32.5-40.0)
No	35.3%(34.5-36.1)	30.0%(29.3-30.8)	34.9%(32.3-37.6)	29.2%(28.4-30.1)	26.6%(26.0-27.2)	27.6%(27.1-28.1)
p value for difference	0.1741	0.8972	0.0922	0.0003	0.0045	<0.0001
Birth weight (g)						
<2500	38.6%(35.2-42.0)	29.3%(25.3-33.5)	34.9%(32.3-37.6)	32.4%(28.5-36.6)	32.3%(28.5-36.3)	32.3%(29.6-35.1)
2500-4000	35.4%(34.6-36.2)	30.2%(29.4-31.0)	32.8%(32.2-33.3)	29.2%(28.3-30.1)	27.0%(26.3-27.7)	27.8%(27.3-28.4)
⩾4000	33.0%(30.1-36.0)	28.5%(25.9-31.2)	30.3%(28.4-32.3)	30.5%(28.4-32.6)	24.9%(23.5-26.3)	26.9%(25.7-28.0)
p value for trend	0.0154	0.4345	0.0036	0.9208	0.0002	0.0030
Parental smoking						
None	34.0%(33.0-35.0)	29.3%(28.3-30.3)	31.6%(30.9-32.3)	27.7%(26.7-28.6)	24.9%(24.2-25.6)	25.9%(25.4-26.5)
One	37.2%(36.0-38.3)	30.9%(29.8-32.0)	33.9%(33.1-34.7)	33.7%(32.1-35.3)	31.4%(30.2-32.6)	32.3%(31.3-33.3)
Two	36.1%(26.9-46.2)	31.9%(21.4-44.0)	34.3%(27.3-41.8)	39.1%(25.2-54.4)	26.4%(16.7-38.2)	31.6%(23.4-40.8)
p value for trend	<0.0001	0.0261	<0.0001	<0.0001	<0.0001	<0.0001
Used antibiotics						
Yes	40.5%(39.6-41.4)	35.0%(34.1-35.9)	37.8%(37.2-38.4)	9.8%(8.8-10.9)	9.3%(8.6-10.1)	9.5%(8.9-10.2)
No	17.2%(15.9-18.5)	15.0%(13.0-16.2)	16.0%(15.2-16.9)	36.2%(35.2-37.2)	33.4%(32.6-34.2)	34.4%(33.9-35.1)
p value for difference	<0.0001	<0.0001	<0.0001	<0.0001	<0.0001	<0.0001
History of parental asthma						
None	35.1%(34.3-35.8)	29.7%(29.0-30.5)	32.3%(31.8-32.9)	29.1%(28.3-30.0)	26.5%(25.8-27.1)	27.5%(27.0-28.0)
One	46.0%(41.7-50.4)	40.8%(35.9-45.9)	43.9%(40.6-47.1)	44.9%(39.0-59.0)	40.2%(35.3-45.4)	42.2%(38.4-46.0)
Two	22.2%(3.3-57.9)	53.2%(23.5-81.3)	49.1%(27.5-71.0)	16.9%(5.9-34.7)	25.2%(14.4-38.9)	22.3%(13.9-32.6)
p value for trend	<0.0001	<0.0001	<0.0001	0.0001	<0.0001	<0.0001

History of parental allergy						
None	34.0%(33.2-34.7)	29.0%(28.2-29.7)	31.4%(30.8-31.9)	26.3%(25.4-27.3)	23.9%(23.2-24.6)	24.8%(24.2-25.3)
One	46.8%(44.4-49.2)	39.9%(37.3-42.6)	43.8%(42.0-45.6)	36.3%(34.7-38.1)	33.3%(32.0-34.6)	34.5%(33.5-35.6)
Two	46.5%(38.8-54.3)	51.9%(43.1-60.6)	48.6%(42.9-54.4)	39.4%(35.3-43.6)	36.0%(32.9-39.2)	37.3%(34.8-39.8)
p value for trend	<0.0001	<0.0001	<0.0001	<0.0001	<0.0001	<0.0001
Residence area						
＜75m²	34.8%(33.6-36.1)	29.6%(28.4-30.7)	32.1%(31.2-32.9)	30.5%(28.9-32.3)	25.2%(24.0-26.5)	27.3%(26.3-28.3)
⩾75m²	35.8%(34.8-36.7)	30.4%(29.4-31.3)	33.1%(32.4-33.8)	29.1%(28.2-30.1)	27.2%(26.5-27.9)	27.9%(27.4-28.5)
p value for difference	0.2411	0.2968	0.0609	0.1534	0.0072	0.2811
Frequency of putting bedding to sunshine						
Never	39.3%(35.0-43.6)	33.0%(28.8-37.4)	36.2%(33.2-39.2)	23.7%(19.7-28.2)	26.3%(22.2-30.7)	25.1%(22.2-28.2)
Sometimes	38.9%(37.5-43.0)	32.7%(31.4-34.0)	35.6%(34.7-36.6)	31.8%(30.3-33.2)	30.1%(29.0-31.2)	30.7%(29.9-31.6)
Often	33.4%(32.5-34.4)	28.6%(27.7-29.5)	31.0%(30.4-31.6)	28.5%(27.5-29.6)	25.0%(24.2-25.7)	26.3%(25.7-26.9)
p value for trend	<0.0001	<0.0001	<0.0001	0.0799	<0.0001	<0.0001
Frequency of clean children's rooms						
Rarely	32.7%(26.5-39.4)	29.7%(23.7-36.2)	30.8%(26.5-35.4)	30.8%(26.0-35.8)	28.9%(24.8-33.2)	29.8%(26.7-33.0)
Sometimes	36.3%(34.4-38.2)	32.4%(30.5-34.3)	34.4%(33.1-35.8)	32.0%(30.1-33.9)	28.9%(27.4-30.4)	30.1%(29.0-31.3)
Often	35.3%(34.5-36.1)	29.6%(28.9-30.4)	32.4%(31.8-33.0)	28.8%(27.9-29.8)	26.2%(25.5-26.9)	27.2%(26.6-27.7)
p value for trend	0.6781	0.0280	0.0533	0.0067	0.0010	<0.0001
Cooking fuel type						
Natural gas/coal gas	35.8%(34.9-36.7)	31.1%(30.3-31.9)	33.4%(32.8-34.0)	29.9%(29.1-30.8)	26.9%(26.3-27.6)	28.1%(27.6-28.6)
Coal	37.4%(34.6-40.3)	29.3%(26.1-32.6)	33.9%(31.8-36.1)	33.6%(20.6-48.7)	39.7%(27.2-53.3)	36.9%(27.8-46.8)
Electricity	30.8%(38.7-32.9)	25.0%(23.1-26.9)	27.6%(26.2-29.0)	23.7%(20.3-27.4)	23.7%(20.9-26.7)	24.0%(21.8-26.2)

Wood	29.8%(20.2-40.9)	24.7%(16.7-34.2)	25.8%(19.7-32.8)	51.1%(18.1-83.4)	0.0%(0.0-52.2)	29.2%(8.7-58.7)
Other	39.8%(36.0-43.7)	30.9%(27.6-34.3)	34.9%(32.4-37.5)	12.6%(6.9-20.4)	9.6%(4.5-17.5)	11.0%(7.0-16.2)
p value for difference	<0.0001	<0.0001	<0.0001	<0.0001	<0.0001	<0.0001
Indoor dampness						
Yes	42.0%(39.9-44.1)	37.0%(34.8-39.1)	39.5%(38.0-41.0)	36.3%(34.0-38.6)	32.4%(30.7-34.2)	33.9%(32.5-35.2)
No	34.4%(33.6-35.2)	29.0%(28.3-29.8)	31.7%(31.1-32.2)	28.4%(27.6-29.3)	25.8%(25.1-26.5)	26.8%(26.3-27.3)
p value for difference	<0.0001	<0.0001	<0.0001	<0.0001	<0.0001	<0.0001
Home interior decoration						
Yes	39.0%(37.2-40.8)	32.7%(31.0-34.6)	35.9%(34.6-37.2)	27.6%(25.1-30.1)	25.3%(23.4-27.2)	26.2%(24.7-27.7)
No	34.7%(33.8-35.5)	29.5%(28.7-30.2)	32.0%(31.4-32.6)	29.7%(28.9-30.6)	26.9%(26.3-27.6)	28.0%(27.4-28.5)
p value for difference	<0.0001	0.0007	0.0001	0.1113	0.1143	0.0265
Home interior purchase new furniture						
Yes	38.2%(36.9-39.5)	31.2%(29.9-32.4)	31.7%(31.0-32.3)	28.7%(27.0-30.5)	26.4%(25.1-27.7)	27.3%(26.2-28.4)
No	34.0%(33.1-34.9)	29.4%(28.5-30.3)	34.6%(33.7-35.5)	29.7%(28.7-30.6)	26.8%(26.1-27.5)	27.9%(27.3-28.4)
p value for difference	<0.0001	0.0227	<0.0001	0.3672	0.6198	0.3308
Wall painting materials						
Wall paper	34.7%(32.4-37.1)	29.5%(27.1-32.0)	32.3%(30.6-34.0)	28.0%(26.4-29.7)	26.8%(25.6-28.1)	27.3%(26.3-28.3)
Emulsion paint	36.3%(35.3-37.3)	31.4%(30.4-32.4)	33.8%(33.1-34.5)	32.0%(30.7-33.3)	27.3%(26.4-28.3)	29.1%(28.3-29.9)
Paint	38.5%(36.3-40.7)	31.9%(29.5-34.4)	35.6%(34.0-37.2)	31.9%(26.5-37.6)	25.1%(21.3-29.2)	27.7%(24.6-31.0)
Wood	20.8%(14.7-28.0)	24.2%(18.1-31.2)	23.0%(18.6-27.9)	14.1%(7.5-23.3)	14.6%(8.8-22.3)	14.5%(10.0-20.1)
Lime / cement	31.7%(29.6-33.8)	26.3%(24.7-28.0)	28.6%(27.3-29.9)	26.4%(21.0-32.5)	23.4%(19.6-27.5)	24.4%(21.3-27.7)
Other	30.8%(27.5-34.3)	27.3%(24.5-30.3)	28.9%(26.7-31.1)	27.6%(26.2-29.1)	26.5%(25.4-27.7)	27.0%(26.1-27.9)
p value for difference	<0.0001	<0.0001	<0.0001	<0.0001	0.0177	<0.0001
Flooring materials						

Solid wood / multi-layer solid wood	34.4%(33.4-35.4)	29.4%(28.3-30.5)	32.1%(31.4-32.9)	29.4%(28.1-30.7)	25.7%(24.7-26.7)	27.2%(26.4-27.9)
Laminate / Composite wood	39.5%(37.9-41.1)	34.5%(32.9-36.1)	37.0%(35.9-38.1)	31.2%(29.8-32.7)	28.9%(27.8-30.1)	29.8%(28.9-30.7)
Bamboo	28.9%(22.9-35.6)	30.8%(25.0-37.1)	30.0%(25.8-34.5)	21.8%(14.4-30.9)	25.1%(18.9-32.2)	23.9%(19.0-29.3)
Ceramic tile / stone / cement	33.7%(32.1-35.4)	28.2%(27.0-29.5)	30.4%(29.4-31.4)	28.8%(27.1-30.5)	25.9%(24.8-27.1)	26.9%(25.9-27.8)
PVC / plastics / plastic leather	35.1%(28.2-42.4)	24.1%(18.4-30.6)	28.9%(24.5-33.7)	21.7%(10.2-37.5)	29.6%(18.7-42.6)	27.9%(19.4-37.6)
Other	32.7%(26.9-38.9)	29.2%(23.9-34.9)	30.9%(26.9-35.0)	20.2%(15.3-25.8)	22.5%(18.1-27.4)	21.7%(18.3-25.3)
p value for difference	<0.0001	<0.0001	<0.0001	0.0011	0.0002	<0.0001
Indoor heating mode						
No heating	33.6%(31.5-35.8)	28.6%(26.7-30.6)	31.0%(29.5-32.4)	29.6%(28.1-31.1)	26.1%(24.9-27.2)	27.5%(26.6-28.4)
Individual household heating	35.0%(34.1-35.9)	29.6%(28.6-30.5)	32.4%(31.8-33.1)	30.2%(29.0-31.4)	27.6%(26.7-28.6)	28.7%(27.9-29.4)
Central heating	38.7%(36.9-40.5)	33.6%(32.1-35.2)	35.7%(34.5-36.9)	27.7%(25.9-29.6)	26.2%(24.9-27.5)	26.7%(25.6-27.7)
Other	37.7%(31.6-44.1)	22.5%(17.9-27.7)	29.1%(25.3-33.1)	28.7%(24.3-33.4)	24.2%(21.2-27.3)	25.7%(23.2-28.3)
p value for difference	0.0007	<0.0001	<0.0001	0.1608	0.0384	0.0052
Indoor use air-conditioning						
Yes	34.8%(33.9-35.6)	29.4%(28.5-30.3)	33.4%(32.5-34.3)	30.0%(29.1-31.0)	26.8%(26.1-27.5)	28.1%(27.5-28.6)
No	36.6%(35.1-38.0)	31.0%(29.8-32.2)	32.2%(31.6-32.8)	27.1%(25.2-29.0)	26.4%(25.0-27.7)	26.6%(25.5-27.7)
p value for difference	0.0347	0.0324	0.0327	0.0065	0.5690	0.0193
Indoor use air purifier						
Yes	36.2%(33.1-39.3)	33.0%(29.4-36.6)	34.7%(32.4-37.1)	30.2%(28.9-31.6)	27.1%(26.1-28.2)	28.3%(27.5-29.1)
No	35.4%(34.6-36.2)	29.9%(29.2-30.7)	32.6%(32.0-33.1)	29.1%(28.1-30.1)	26.6%(25.8-27.4)	27.6%(26.9-28.2)
p value for difference	0.6361	0.0969	0.0780	0.1957	0.4028	0.1681
Indoor keeping furry pets						
Yes	35.5%(33.2-37.8)	29.0%(27.0-31.1)	32.1%(30.6-33.7)	31.2%(28.8-33.7)	28.4%(26.6-30.3)	29.4%(28.0-30.9)

	No	35.5%(33.2-37.8)	30.2%(29.4-31.0)	32.8%(32.3-33.4)	29.3%(28.4-30.1)	26.5%(25.9-27.2)	27.6%(27.1-28.1)
p value for difference		0.9918	0.3070	0.4220	0.1485	0.0607	0.0179
Asthma							
	Yes	62.6%(60.1-65.1)	61.6%(58.5-64.6)	62.2%(60.3-64.1)	59.0%(54.8-63.1)	59.8%(56.0-63.6)	59.5%(56.6-62.2)
	No	32.8%(32.0-33.6)	28.0%(27.2-28.7)	30.3%(29.8-30.8)	28.0%(27.2-28.9)	25.6%(25.0-26.2)	26.5%(26.0-27.0)
p value for difference		<0.0001	<0.0001	<0.0001	<0.0001	<0.0001	<0.0001
Allergic rhinitis							
	Yes	48.9%(46.5-51.2)	46.0%(43.2-48.7)	47.6%(45.9-49.4)	46.1%(43.7-48.6)	42.4%(40.3-44.6)	44.1%(42.5-45.7)
	No	33.7%(32.9-34.5)	28.7%(27.9-29.4)	31.1%(30.6-31.6)	26.9%(26.1-27.8)	24.9%(24.3-25.6)	25.6%(25.1-26.2)
p value for difference		<0.0001	<0.0001	<0.0001	<0.0001	<0.0001	<0.0001
Wheezing							
	Yes	50.0%(48.5-51.6)	44.4%(42.8-45.9)	47.1%(46.0-48.2)	58.3%(55.2-61.3)	58.3%(55.2-61.3)	57.3%(55.2-59.4)
	No	30.7%(29.8-31.5)	26.2%(25.4-27.0)	28.4%(27.8-29.0)	26.8%(26.0-27.6)	26.8%(26.0-27.6)	25.6%(25.1-26.1)
p value for difference		<0.0001	<0.0001	<0.0001	<0.0001	<0.0001	<0.0001

Values are % (95% CI). p value for difference refers to the comparison of binary variables. Pneumonia, asthma, allergic rhinitis were defined as physician-diagnosed since birth of children. Wheezing was defined as parents reported that the child has great difficulty breathing or the respiratory muscles of the child are all involved in breathing, and the respiratory rate of the child is faster than normal since birth. Premature birth was defined as delivery under 37 weeks of pregnancy. Used antibiotics was defined as child has been injected or taken since birth, such as but not limited to penicillin, azithromycin, cephalosporin, etc. Parental smoking was defined as parents smoked equal to or more than 100 cigarettes in the lifetime. All calculations of p values are weighted, accounting for the number of people sampled in two surveys in 2011 and 2019 and based on the χ^2 test.

Table S5: Age-specific and age-adjusted prevalence of pneumonia among preschool children in the Chinese seven cities in 2011 and 2019 by antibiotics use

	2011			2019		
	Never used	Used antibiotics	Total	Never used	Used antibiotics	Total

		antibiotics			antibiotics		
Age (years)							
	2-4	15.2%(12.9-17.7)	36.4%(34.7-38.1)	31.7%(30.3-33.2)	33.7%(32.5-35.0)	10.2%(9.0-11.6)	27.5%(26.4-28.5)
	4-6	17.3%(16.2-18.4)	39.5%(38.7-40.3)	34.3%(33.6-35.0)	34.7%(33.9-35.5)	9.0%(8.2-9.8)	27.9%(27.3-28.5)
	6-8	15.6%(14.0-17.3)	37.5%(36.2-38.7)	32.0%(31.0-33.1)	35.0%(33.5-36.4)	9.3%(7.9-10.9)	28.0%(26.8-29.2)
p value for trend		0.7255	0.8968	0.4410	0.1857	0.2672	0.4490
Gender							
	Boys	16.8%(15.6-18.0)	39.8%(39.0-40.7)	34.6%(33.8-35.3)	36.2%(35.4-37.1)	10.1%(9.2-11.0)	29.3%(28.6-30.0)
	Girls	15.2%(14.1-16.4)	35.5%(34.7-36.4)	30.6%(29.9-31.4)	32.5%(31.7-33.4)	8.9%(8.0-9.8)	26.1%(25.4-26.8)
p value for difference		0.0682	<0.0001	<0.0001	<0.0001	0.0641	<0.0001
Urbanisation							
	Urban	16.2%(15.2-17.2)	38.7%(38.0-39.5)	33.7%(33.1-34.3)	34.0%(33.4-34.7)	9.3%(8.6-10.0)	27.6%(27.1-28.2)
	Suburb	15.8%(14.1-17.7)	35.4%(34.1-36.8)	30.6%(29.4-31.7)	36.5%(34.9-38.1)	10.6%(9.0-12.3)	29.2%(27.9-30.5)
	Rural	13.2%(10.1-16.8)	30.5%(27.0-34.1)	23.6%(21.1-26.2)	36.1%(32.5-39.9)	9.6%(7.0-12.7)	25.6%(23.0-28.2)
p value for difference		0.2712	<0.0001	<0.0001	0.0108	0.3197	0.0169
Duration of breastfeeding							
	＜6 months	17.2%(15.9-18.5)	40.5%(39.6-41.4)	35.4%(34.7-36.2)	36.2%(35.2-37.2)	9.8%(8.7-10.9)	29.5%(28.7-30.3)
	⩾6 months	15.0%(13.9-16.2)	35.0%(34.1-35.9)	30.0%(29.3-30.8)	33.4%(32.6-34.2)	9.3%(8.6-10.1)	26.7%(26.1-27.4)
p value for difference		0.0117	<0.0001	<0.0001	<0.0001	0.5074	<0.0001
Premature birth							
	Yes	16.0%(12.2-20.5)	41.2%(38.1-44.4)	32.6%(32.1-33.1)	44.0%(39.6-48.5)	11.0%(6.4-17.1)	36.2%(32.5-40.0)
	No	16.0%(15.2-16.9)	37.6%(37.0-38.3)	34.9%(32.3-37.6)	34.3%(33.6-34.9)	9.5%(8.7-10.1)	27.6%(27.1-28.1)
p value for difference		1.0000	0.0278	0.0922	<0.0001	0.6409	<0.0001
Birth weight (g)							
	<2500	15.7%(11.8-20.2)	41.2%(38.1-44.3)	34.9%(32.3-37.6)	41.8%(38.3-45.2)	8.5%(5.7-12.1)	32.3%(29.6-35.1)

2500-4000	16.0%(15.1-16.9)	37.9%(37.2-38.5)	32.8%(32.2-33.3)	34.3%(33.6-35.0)	9.5%(8.8-10.2)	27.8%(27.3-28.4)
≥4000	16.3%(13.2-19.8)	34.6%(32.2-36.9)	30.3%(28.4-32.3)	34.0%(32.5-35.5)	9.7%(8.3-11.3)	26.9%(25.7-28.0)
p value for trend	0.8207	0.0005	0.0036	0.0111	0.5785	0.0030
Parental smoking						
None	14.4%(13.4-15.6)	36.8%(36.0-37.6)	31.6%(30.9-32.3)	32.6%(31.9-33.3)	8.5%(7.8-9.2)	25.9%(25.4-26.5)
One	17.9%(16.6-19.3)	38.9%(38.0-39.8)	33.9%(33.1-34.7)	38.9%(37.7-40.1)	12.2%(10.8-13.6)	32.3%(31.3-33.3)
Two	13.1%(5.3-25.3)	42.8%(33.9-52.0)	34.3%(27.3-41.8)	36.7%(26.3-48.0)	21.4%(9.5-38.2)	31.6%(23.4-40.8)
p value for trend	0.0002	0.0004	<0.0001	<0.0001	<0.0001	<0.0001
History of parental asthma						
None	15.7%(14.9-16.6)	37.5%(36.9-38.1)	32.3%(31.8-32.9)	34.2%(33.5-34.8)	9.4%(8.8-10.0)	27.5%(27.0-28.0)
One	31.6%(24.1-39.8)	46.2%(42.6-49.8)	43.9%(40.6-47.1)	47.7%(43.4-52.0)	17.4%(11.1-25.2)	42.2%(38.4-46.0)
Two	0(0-60.2)	53.7%(29.2-77.0)	49.1%(27.5-71.0)	24.3%(15.2-35.6)	5.6%(0.0-38.9)	22.3%(13.9-32.6)
p value for trend	<0.0001	<0.0001	<0.0001	<0.0001	0.0165	<0.0001
History of parental allergy						
None	15.2%(14.4-16.1)	36.6%(36.0-37.3)	31.4%(30.8-31.9)	31.7%(31.0-32.4)	8.8%(8.2-9.5)	24.8%(24.2-25.3)
One	27.3%(22.8-32.2)	46.1%(44.1-48.0)	43.8%(42.0-45.6)	39.8%(38.6-41.0)	11.3%(9.7-13.0)	34.5%(33.5-35.6)
Two	36.9%(22.7-52.9)	50.7%(44.5-56.9)	48.6%(42.9-54.4)	41.5%(38.7-44.3)	16.8%(12.3-22.0)	37.3%(34.8-39.8)
p value for trend	<0.0001	<0.0001	<0.0001	<0.0001	<0.0001	<0.0001
Residence area						
$<75m^2$	15.8%(14.5-17.1)	37.5%(36.5-38.5)	32.1%(31.2-32.9)	33.5%(32.3-34.8)	10.3%(9.0-11.7)	27.3%(26.3-28.3)
$\geq 75m^2$	16.2%(15.1-17.3)	37.9%(37.1-38.7)	33.1%(32.4-33.8)	34.7%(34.0-35.5)	9.3%(8.6-10.0)	27.9%(27.4-28.5)
p value for difference	0.7040	0.5652	0.0609	0.1009	0.1722	0.2811
Frequency of putting bedding to sunshine						
Never	20.3%(15.5-25.7)	41.5%(37.9-45.1)	36.2%(33.2-39.2)	37.1%(32.8-41.6)	9.0%(6.3-12.5)	25.1%(22.2-28.2)

Sometimes	18.0%(16.3-19.8)	40.1%(39.0-41.2)	35.6%(34.7-36.6)	36.7%(35.7-37.7)	11.4%(10.2-12.7)	30.7%(29.9-31.6)
Often	14.9%(14.0-16.0)	36.3%(35.6-37.1)	31.0%(30.4-31.6)	33.1%(32.3-33.8)	8.7%(7.9-9.4)	26.3%(25.7-26.9)
p value for trend	0.0002	<0.0001	<0.0001	<0.0001	0.0032	<0.0001
Frequency of clean children's rooms						
Rarely	11.0%(6.0-18.0)	39.0%(33.6-44.6)	30.8%(26.5-35.4)	36.6%(32.8-40.5)	8.3%(4.8-13.1)	29.8%(26.7-33.0)
Sometimes	14.8%(12.6-17.2)	39.1%(37.5-40.6)	34.4%(33.1-35.8)	36.0%(34.6-37.4)	10.2%(8.6-11.9)	30.1%(29.0-31.3)
Often	16.3%(15.4-17.3)	37.5%(36.8-38.2)	32.4%(31.8-33.0)	34.0%(33.3-34.7)	9.5%(8.8-10.2)	27.2%(26.6-27.7)
p value for trend	0.0669	0.0659	0.0533	0.0084	0.8033	<0.0001
Cooking fuel type						
Natural gas/coal gas	16.3%(15.3-17.4)	38.3%(37.6-39.0)	33.4%(32.8-34.0)	34.6%(34.0-35.2)	9.6%(9.0-10.3)	28.1%(27.6-28.6)
Coal	15.5%(12.5-19.1)	40.3%(37.8-42.9)	33.9%(31.8-36.1)	45.3%(33.1-58.0)	25.1%(12.7-41.3)	36.9%(27.8-46.8)
Electricity	15.0%(13.1-17.2)	32.9%(31.1-34.6)	27.6%(26.2-29.0)	32.3%(29.4-35.4)	7.8%(5.6-10.5)	24.0%(21.8-26.2)
Wood	13.5%(6.2-24.5)	33.5%(25.2-42.7)	25.8%(19.7-32.8)	38.9%(9.2-76.6)	0.0%(0.0-45.9)	29.2%(8.7-58.7)
Other	15.6%(11.8-20.0)	40.4%(37.4-43.3)	34.9%(32.4-37.5)	18.2%(10.5-28.2)	5.9%(2.4-11.8)	11.0%(7.0-16.2)
p value for difference			<0.0001	0.0041	0.0038	<0.0001
Indoor dampness						
Yes	21.2%(18.3-24.3)	43.5%(41.8-45.2)	39.5%(38.0-41.0)	39.6%(38.0-41.2)	11.4%(9.5-13.6)	33.9%(32.5-35.2)
No	15.4%(14.6-16.3)	36.9%(36.2-37.5)	31.7%(31.1-32.2)	33.5%(32.9-34.2)	9.3%(8.6-9.9)	26.8%(26.3-27.3)
p value for difference	<0.0001	<0.0001	<0.0001	<0.0001	0.0349	<0.0001
Home interior decoration						
Yes	16.9%(14.7-19.4)	40.3%(38.8-41.7)	35.9%(34.6-37.2)	32.2%(30.4-34.1)	9.0%(7.2-11.1)	26.2%(24.7-27.7)
No	15.8%(14.9-16.7)	37.2%(36.5-37.9)	32.0%(31.4-32.6)	34.7%(34.1-35.4)	9.5%(8.9-10.2)	28.0%(27.4-28.5)
p value for difference	0.3613	0.0002	0.0001	0.0128	0.6091	0.0265
Home interior purchase new furniture						

Yes	17.6%(16.0-19.2)	38.8%(37.8-39.9)	31.7%(31.0-32.3)	33.4%(32.1-34.7)	8.8%(7.5-10.2)	27.3%(26.2-28.4)
No	15.4%(14.4-16.4)	37.2%(36.4-37.9)	34.6%(33.7-35.5)	34.7%(34.0-35.4)	9.6%(9.0-10.4)	27.9%(27.3-28.4)
p value for difference	0.0234	0.0102	<0.0001	0.0803	0.2884	0.3308
Wall painting materials						
Wall paper	14.4%(12.0-17.2)	38.1%(36.1-40.1)	32.3%(30.6-34.0)	34.4%(33.1-35.6)	9.1%(7.9-10.4)	27.3%(26.3-28.3)
Emulsion paint	17.2%(16.0-18.5)	38.3%(37.5-39.1)	33.8%(33.1-34.5)	34.5%(33.6-35.5)	9.5%(8.4-10.6)	29.1%(28.3-29.9)
Paint	15.9%(13.4-18.7)	40.9%(39.1-42.8)	35.6%(34.0-37.2)	35.1%(31.1-39.3)	11.0%(7.9-10.4)	27.7%(24.6-31.0)
Wood	6.5%(2.9-12.2)	34.0%(27.4-41.0)	23.0%(18.6-27.9)	22.7%(15.0-32.1)	5.6%(2.1-12.0)	14.5%(10.0-20.1)
Lime / cement	15.2%(13.3-17.2)	34.0%(32.4-35.6)	28.6%(27.3-29.9)	33.2%(28.7-37.8)	10.7%(7.3-15.0)	24.4%(21.3-27.7)
Other	14.0%(11.1-17.3)	35.8%(33.0-38.6)	28.9%(26.7-31.1)	34.6%(33.5-35.8)	9.8%（8.8-11.0）	27.0%(26.1-27.9)
p value for difference	0.0055	<0.0001	<0.0001	0.2389	0.6140	<0.0001
Flooring materials						
Solid wood / multi-layer solid wood	16.1%(14.9-17.3)	37.1%(36.2-38.0)	32.1%(31.4-32.9)	34.3%(33.3-35.3)	9.0%（8.0-10.0）	27.2%(26.4-27.9)
Laminate / Composite wood	19.0%(16.8-21.3)	40.7%(39.4-42.0)	37.0%(35.9-38.1)	35.7%(34.6-36.7)	9.4%（8.3-10.7）	29.8%(28.9-30.7)
Bamboo	16.3%(9.7-25.0)	33.5%(28.5-38.8)	30.0%(25.8-34.5)	31.1%(24.5-38.2)	9.9%（4.7-17.9）	23.9%(19.0-29.3)
Ceramic tile / stone / cement	14.8%(13.3-16.4)	36.2%(35.0-37.4)	30.4%(29.4-31.4)	33.6%(32.4-34.8)	10.5%（9.3-11.8）	26.9%(25.9-27.8)
PVC / plastics / plastic leather	22.3%(14.5-32.0)	31.4%(36.1-37.0)	28.9%(24.5-33.7)	41.3%(29.3-54.9)	4.8%(1.0-16.7)	27.9%(19.4-37.6)
Other	12.7%(8.3-18.3)	40.5%(35.2-46.0)	30.9%(26.9-35.0)	27.6%(23.1-32.5)	10.3%(4.5-15.4)	21.7%(18.3-25.3)
p value for difference	0.0137	<0.0001	<0.0001	0.0041	0.4183	<0.0001
Indoor heating mode						
No heating	16.2%(14.0-18.5)	36.2%(34.4-37.9)	31.0%(29.5-32.4)	34.4%(33.3-35.6)	8.1%(7.0-9.2)	27.5%(26.6-28.4)
Individual household heating	16.5%(15.4-17.6)	37.2%(36.4-37.9)	32.4%(31.8-33.1)	34.5%(33.6-35.4)	10.3%(9.3-11.3)	28.7%(27.9-29.4)
Central heating	16.5%(14.6-18.4)	41.4%(40.0-42.7)	35.7%(34.5-36.9)	34.4%(33.1-35.7)	9.5%(8.3-10.8)	26.7%(25.6-27.7)
Other	9.9%(5.9-15.2)	37.5%(32.5-42.6)	29.1%(25.3-33.1)	34.5%(31.1-38.0)	10.6%(7.8-14.0)	25.7%(23.2-28.3)
p value for difference	0.1347	<0.0001	<0.0001	0.9986	0.0290	0.0052

Indoor use air-conditioning							
	Yes	15.6%(14.6-16.7)	36.9%(36.1-37.6)	33.4%(32.5-34.3)	34.2%(33.5-34.9)	9.5%(8.7-10.2)	28.1%(27.5-28.6)
	No	16.8%(15.4-18.3)	39.4%(38.3-40.6)	32.2%(31.6-32.8)	35.7%(34.2-37.1)	9.6%(8.4-10.9)	26.6%(25.5-27.7)
p value for difference		0.1636	0.0001	0.0327	0.0765	0.8745	0.0193
Indoor use air purifier							
	Yes	19.0%(15.0-23.6)	38.9%(36.3-41.6)	34.7%(32.4-37.1)	35.4%(34.4-36.4)	8.2%(7.3-9.2)	28.3%(27.5-29.1)
	No	15.9%(15.0-16.7)	37.7%(37.1-38.3)	32.6%(32.0-33.1)	34.0%(33.2-34.8)	10.2%(9.5-11.1)	27.6%(26.9-28.2)
p value for difference		0.1352	0.3988	0.0780	0.0293	0.0023	0.1681
Indoor keeping furry pets							
	Yes	19.5%(16.9-22.1)	36.4%(34.6-38.3)	32.1%(30.6-33.7)	35.6%(33.8-37.4)	11.1%(9.2-13.3)	29.4%(28.0-30.9)
	No	15.6%(14.7-16.5)	38.0%(37.3-38.6)	32.8%(32.3-33.4)	34.3%(33.7-35.0)	9.3%%(8.7-10.0)	27.6%(27.1-28.1)
p value for difference		0.0033	0.1256	0.4220	0.2041	0.0773	0.0179
Asthma							
	Yes	52.6%(46.4-58.7)	63.4%(61.4-65.4)	62.2%(60.3-64.1)	61.4%(58.4-64.3)	42.6%(33.5-52.1)	59.5%(56.6-62.2)
	No	14.8%(14.0-15.7)	35.3%(34.7-35.9)	30.3%(29.8-30.8)	33.1%(32.5-33.8)	9.0%(8.4-10.0)	26.5%(26.0-27.0)
p value for difference		<0.0001	<0.0001	<0.0001	<0.0001	<0.0001	<0.0001
Allergic rhinitis							
	Yes	26.4%(22.0-31.1)	50.6%(48.8-52.5)	47.6%(45.9-49.4)	47.3%(45.6-49.0)	19.8%(16.2-23.8)	44.1%(42.5-45.7)
	No	15.5%(14.7-16.4)	36.2%(35.5-36.8)	31.1%(30.6-31.6)	32.4%(31.7-33.0)	9.0%(8.4-9.6)	25.6%(25.1-26.2)
p value for difference		<0.0001	<0.0001	<0.0001	<0.0001	<0.0001	<0.0001
Wheezing							
	Yes	29.4%(26.9-32.0)	50.3%(49.1-51.5)	47.1%(46.0-48.2)	60.2%(58.0-62.3)	31.5%(25.2-38.4)	57.3%(55.2-59.4)
	No	13.9%(13.0-14.8)	33.5%(32.8-34.2)	28.4%(27.8-29.0)	32.0%(31.4-32.7)	9.0%(8.3-9.6)	25.6%(25.1-26.1)
p value for difference		<0.0001	<0.0001	<0.0001	<0.0001	<0.0001	<0.0001

Values are % (95% CI). p value for difference refers to the comparison of binary variables. Pneumonia, asthma, allergic rhinitis were defined as

physician-diagnosed since birth of children. Wheezing was defined as parents reported that the child has great difficulty breathing or the respiratory muscles of the child are all involved in breathing, and the respiratory rate of the child is faster than normal since birth. Premature birth was defined as delivery under 37 weeks of pregnancy. Used antibiotics was defined as child has been injected or taken since birth, such as but not limited to penicillin, azithromycin, cephalosporin, etc. Parental smoking was defined as parents smoked equal to or more than 100 cigarettes in the lifetime. All calculations of p values are weighted, accounting for the number of people sampled in two surveys in 2011 and 2019 and based on the χ^2 test.
